# Entomological determinants of malaria transmission in Kayin state, Eastern Myanmar: A 24-month longitudinal study in four villages

**DOI:** 10.12688/wellcomeopenres.14761.4

**Published:** 2019-06-17

**Authors:** Victor Chaumeau, Bénédicte Fustec, Saw Nay Hsel, Céline Montazeau, Saw Naw Nyo, Selma Metaane, Sunisa Sawasdichai, Prapan Kittiphanakun, Phabele Phatharakokordbun, Nittipha Kwansomboon, Chiara Andolina, Dominique Cerqueira, Theeraphap Chareonviriyaphap, François H. Nosten, Vincent Corbel

**Affiliations:** 1 Centre Hospitalier Universitaire de Montpellier, Montpellier, 34295, France; 2 Maladies Infectieuses et Vecteurs, Ecologie, Génétique, Evolution et Contrôle, Institut de Recherche pour le Développement, Montpellier, 34394, France; 3 Shoklo Malaria Research Unit, Mahidol-Oxford Tropical Medicine Research Unit, Faculty of Tropical Medicine, Mahidol University, Mae Sot, 63110, Thailand; 4 Centre for Tropical Medicine and Global Health, Nuffield Department of Medicine, University of Oxford, Oxford, OX3 7BN, UK; 5 Department of Entomology, Faculty of Agriculture, Kasetsart University, Bangkok, 10900, Thailand

**Keywords:** Anopheles, human biting rate, sporozoite index, entomological inoculation rate, parasite load, residual transmission, Plasmodium juxtanucleare, zoophagy, exophagy, hypnozoite reservoire

## Abstract

**Background**: The Thailand-Myanmar borderland is an area endemic for malaria where transmission is low, seasonal and unstable. The epidemiology has been described but there is relatively few data on the entomological determinants of malaria transmission.

**Methods**: Entomological investigations were conducted during 24 months in four villages located in Kayin state, on the Myanmar side of the Thailand-Myanmar border. *Anopheles* mosquitoes were identified by morphology, and molecular assays were used in order to discriminate between closely related sibling species of malaria vectors. *Plasmodium* infection rate was determined using quantitative real-time PCR.

**Results**: The diversity of *Anopheles* mosquitoes was very high and multiple species were identified as malaria vectors. The intensity of human-vector contact (mean human-biting rate= 369 bites/person/month) compensates for the low infection rate in naturally infected populations of malaria vectors (mean sporozoite index= 0.04 and 0.17 % for *P. falciparum* and *P. vivax* respectively), yielding intermediary level of transmission intensity (mean entomological inoculation rate= 0.13 and 0.64 infective bites/person/month for *P. falciparum* and *P. vivax,* respectively). Only 36% of the infected mosquitoes were collected indoors between 09:00 pm and 05:00 am, suggesting that mosquito bed-nets would fail to prevent most of the infective bites in the study area.

**Conclusion**: This study provided a unique opportunity to describe the entomology of malaria in low transmission settings of Southeast Asia. Our data are important in the context of malaria elimination in the Greater Mekong Subregion.

## Introduction

Artemisinin resistance in *Plasmodium falciparum* has emerged and spread in the Greater Mekong Sub-region (GMS) ^1^, leading to the failure of several artemisinin-based combination therapies (ACTs) ^2^. Multi-drug resistant parasites spreading from Western Cambodia are responsible for a recent resurgence of the disease across the eastern part of the GMS ^3^. Meanwhile in Myanmar (western GMS), the incidence of clinical malaria cases has declined ^4^. In this area, dihydroartemisinin-piperaquine and artemether-lumefantrine remain effective against *P. falciparum*. It is therefore urgent to eliminate falciparum malaria in Myanmar, the main gateway to India and Bangladesh, before these two ACTs also fall to resistance.

Entomological aspects of malaria transmission are important in the context of elimination as they largely determine intervention design and outcome. For example, the interest of treating asymptomatic infections with mass drug administration or mass screening and treatment obviously depends on the contribution of asymptomatic carriers to the transmission ^5, 6^. Moreover, the efficacy of vector-control interventions such as mass distribution of long-lasting insecticide-impregnated mosquito bed-nets (LLINs) and indoor residual spraying (IRS) campaigns is greatly influenced by the ecology of malaria vectors ^7– 9^.

The transmission of *P. falciparum* is low, seasonal and unstable in Kayin state (Eastern Myanmar) ^10^. Some entomological surveys were conducted previously ^11– 15^, most of them on the Thai side of the Thailand-Myanmar border, where the transmission of *P. falciparum* is now interrupted. In this area, the primary vectors are *Anopheles minimus* ( *s.s.*) (Minimus Complex, Funestus Group), *An. maculatus* ( *s.s.*), *An. sawadwongporni* (Maculatus Group), *An. dirus* ( *s.s.*) and *An. baimaii* (Dirus Complex, Leucosphyrus Group) ^11, 13, 15^. *Anopheles pseudowillmori* (Maculatus Group) ^16^, *An. aconitus* ( *s.s.*) (Funestus Group) ^17^ and some members in the Annularis and Barbirostris Groups ^13^ also play a secondary role in the transmission ( Table 1). Numerous aspects of malaria vector ecology and biology have not been documented and the characteristics of the entomological indices are not known precisely ^18^.

**Table 1.** Vectorial status of the *Anopheles* species most frequently collected on the Thailand-Myanmar border.

Group	Subgroup	Complex	Species	Vectorial status on the TMB
Annularis			*An. annularis* ( *s.l.*)	secondary vector
Barbirostris			*An. barbirostris* ( *s.l.*)	secondary vector †
Funestus	Minimus	Minimus	*An. minimus* ( *s.s.*)	primary vector
			*An. harrisoni*	unknown
	Aconitus		*An. aconitus* ( *s.s.*)	secondary vector
			*An. pampanai*	unknown
			*An. varuna*	unknown
	Culicifacies	Culicifacies	*An. culicifacies* ( *s.l.*)	non vector ‡
Hyrcanus			*An. hyrcanus* ( *s.l.*)	non vector ‡
Jamesii			*An. jamesii* ( *s.l.*)	non vector ‡
Kochi			*An. kochi*	non vector
Leucosphyrus	Leucosphyrus	Dirus	*An. dirus* ( *s.s.*)	primary vector
			*An. baimaii*	primary vector
			*An. cracens*	unknown
			*An. nemophilous*	unknown
			*An. scanloni*	unknown
Maculatus	Maculatus		*An. maculatus* ( *s.s.*)	primary vector
			*An. dravidicus*	unknown
	Sawadwongporni		*An. sawadwongporni*	primary vector
			*An. notanandai*	unknown
			*An. rampae*	unknown
	Unclassified		*An. willmori*	unknown
			*An. pseudowillmori*	secondary vector
Subpictus			*An. subpictus* ( *s.l.*)	non vector ‡
Tessellatus			*An. tessellatus*	non vector ‡

† *Plasmodium vivax* ‡ Some species in these Groups are efficient malaria vectors elsewhere, although they were never found infected with human malaria parasites in the Thailand-Myanmar border area ( *e.g. An. culicifacies* A, *An. sinensis*, *An. subpictus* (s.s.), *An. splendidus* and *An. tessellatus*) ^18, 21, 22^.

Large-scale deployment of community-wide access to early diagnosis and treatments has paved the way for the elimination of falciparum malaria in Kayin state. Mass antimalarial drug administration campaigns were used as an accelerator to elimination in villages with submicroscopic reservoirs of asymptomatic malaria infections ^6, 19, 20^. The objective of this study was to describe the entomological determinants of malaria transmission in Kayin state in order to guide policy making for malaria elimination.

## Methods

### Study villages

**Figure 1.**
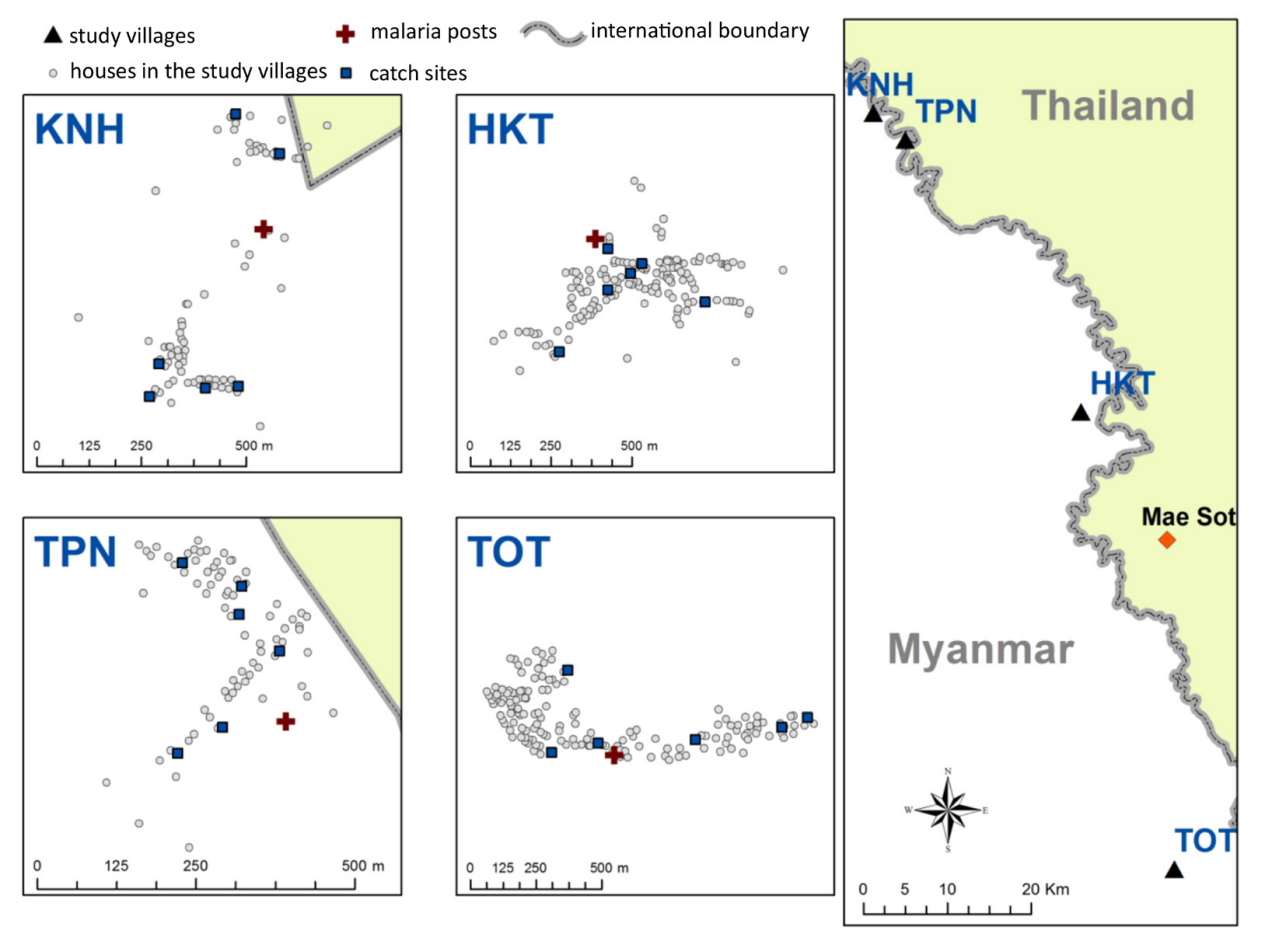
Map of the study sites.

Four villages located in Kayin state were included in the study, namely HKT (16° 85 'N, 98° 47' E), KNH (17° 18 'N, 98° 24' E), TPN (17° 14' N, 98 ° 29' E) and TOT (16° 36' N, 98° 57' E) ( Figure 1). The demographics and baseline malaria epidemiology in the study villages were described previously ^6, 15, 19^. Briefly, the number of households at baseline was 160, 81, 138, and 69 in HKT, KNH, TOT and TPN respectively. The census population at baseline was 908, 349, 745 and 375 at HKT, KNH, TOT and TPN respectively. Residents were mainly farmers and forest workers. The prevalence of submicroscopic malaria at the beginning of the study ranged between 4 and 22% and between 19 and 33% for *P. falciparum* and *P. vivax* respectively.

### Intervention

Community-wide access to early diagnosis and treatments was deployed in all villages for the entire period of the study. Mass antimalarial drug administration campaigns with dihydroartemisinin-piperaquine and a single low dose of primaquine were repeated at monthly intervals for 3 months from 12 June to 24 August 2013 in KNH, from 27 May to 07 August 2013 in TOT, from 28 January to 29 March 2014 in TPN and from 01 April to 10 June 2014 in HKT. LLINs were also distributed to all villagers. The intervention and its impact on the parasitological and entomological indices of malaria were described into more details in Chaumeau *et al.* ^6^ and Landier *et al.* ^19^.

### Entomological surveys

Villages were surveyed monthly from 2013 to 2015 for a total of 21 surveys per village. Entomological surveys consisted of five consecutive nights of collection from 06:00 pm to 06:00 am in five houses per village and on one cow, as described previously ^15^. There were two exceptions to the initial study design: the eleventh survey in TPN was cancelled and mosquitoes were collected for only two nights during the second survey in HKT ( Supplementary File 1, Table S1). In each village, five traditional houses were randomly selected for mosquito sampling using the human-landing catch (HLC) collection method. In each house, one mosquito collector sat indoors and one mosquito collector sat outdoors, yielding a total of 50 person-nights of collection per survey (25 person-nights indoors and 25 person-nights outdoors). Collectors were asked to collect every mosquito landing on their uncovered legs for 50 min per hour and allowed to rest for 10 min per hour. A cow-bait trap (CBT) was also set-up yielding an additional 5 cow-nights of collection. One cow was isolated from the herd and a 1m-wide mosquito net was fenced around the animal, 30cm above the ground level. One collector was asked to collect mosquitoes resting on the net for 50 min per hour and allowed to rest for 10 min. Mosquitoes were shipped at the Shoklo Malaria Research Unit (Mae Sot, Thailand) at the end of each night of collection.

### Laboratory procedures for the processing of entomological samples

Mosquitoes were immediately identified at the genus level by morphology and *Anopheles* specimens were stored individually at -20°C in 1.5 mL plastic tubes containing silica gel. *Anopheles* mosquitoes were identified at the Group or Complex level using the key developed by Rattanarithikul *et al.* ^23^. Deoxyribonucleic acid (DNA) was extracted from head/thorax using a cetyltrimethyl ammonium bromide-based method described previously ^24^. Sibling species to the Funestus, Maculatus and Leucosphyrus Groups were discriminated using allele-specific polymerase chain reaction (AS-PCR) assays adapted from Garros *et al.* and Walton *et al.* ^25– 27^. In case AS-PCR yielded a negative result, identification at the species level was performed by sequencing the internal transcribed spacer 2 (ITS2) DNA marker using universal primers described by Beebe and Saul ^28^. DNA extracts were screened for the presence of *Plasmodium* sporozoites using a quantitative real-time PCR (qPCR) assay targeting 18S rRNA genes adapted from Mangold *et al.* ^29^. Specificity of the signal was confirmed in all positive samples by amplifying COX genes with primers described by Cunha *et al.* ^30^. In case the confirmation assay yielded a negative result, the PCR product of the screening assay was sequenced (BioSample accessions: SAMN09845988, SAMN09845989, SAMN09845990, SAMN09845991, SAMN09845992). The presence of *Plasmodium* oocysts in the abdomen of sporozoites-positive specimens was assessed using the same procedures. Abdomen were preserved at -20°C in 1.5 mL plastic tubes containing silica gel during the time between the screening of head/thorax for sporozoites and the processing of abdomen from sporozoite-positive specimens. The validation of the qPCR assays used for *Plasmodium* detection in this study was published elsewhere ^24^. Detailed laboratory procedures are presented in the Supplementary File 2.

### Data analysis

HBR and CBR were defined as the number of mosquitoes collected divided by the number of person-nights or cow-nights respectively. Results were expressed as a number of bites/person/month or bites/cow/month. Sporozoite index (SI) was defined as the number of mosquitoes positive in qPCR *Plasmodium* divided by the total number of mosquitoes analyzed. Results were expressed as a percentage. Entomological inoculation rate (EIR) was defined as the number of specimens positive in qPCR *Plasmodium* divided by the corresponding number of person-nights of collection ^31^. The number of person-nights used for the calculation of EIR was multiplied by the proportion of collected mosquitoes that were analyzed with qPCR *Plasmodium* in order to take into account that not all mosquitoes were analysed by qPCR ^6, 19^. Results were expressed as a number of infective bites/person/month. The exophagy index (EI) was defined as the outdoor HBR divided by the sum of the indoor and outdoor HBRs. The cow-biting index (CBI) was defined as CBR divided by the sum of the HBR and the CBR. Confidence Intervals (CIs) for Poisson counts (HBR, CBR and EIR) were estimated using the exact method of the poisson.conf.int() function in the *epitools* package version 05-10 of R software version 3.3. Binomial CIs were estimated for proportions (SI, EI and CBI) using the exact method of the binom.confint() function in the *binom* package version 1.1-1 of R software version 3.3. Species-specific proportions were used to compute HBR, CBR, EI and CBI estimates at the species level if ≥30 samples in the corresponding *Anopheles* Group were genotyped for each value of the grouping variable. The grouping variables were the collection time (06:00 pm to 06:00 am), the collection method (HCL or CBT) or the location of the mosquito collector (indoors or outdoors). For example, the number of *An. maculatus* ( *s.l.*) collected by HLC indoors and outdoors was 2240 and 5041 respectively. The proportion of *An. maculatus* ( *s.s.*) in the indoor and outdoor populations was 0.34 (133/392) and 0.37 (404/1084) respectively. The predicted number of *An. maculatus* ( *s.s.*) collected by HLC indoors and outdoors was 762 and 1865 respectively. The EI estimate for *An. maculatus* ( *s.s.*) was therefore 0.71 (1865/2627).

### Ethics approval

The protocol for mosquito collection and analysis has been approved by the Oxford Tropical Research Ethics Committee (1015–13, dated 29 Apr 2013) and by the Ethics Review Committee for Research Involving Human Research Subjects, Health Science Group, Chulalongkorn University (COA 154/2014). All participants provided their written consent to participate in this study. This consent procedure was approved by the ethics committees.

## Results

### *Anopheles* diversity

In total, 129228 *Anopheles* mosquitoes were collected during 4120 person-nights and 412 cow-nights of collection (63217 by HLC and 66011 by CBT). We report the occurrence of 10 Groups of *Anopheles* on the basis of morphological identification: Barbirostris, Hyrcanus ( *Anopheles* Subgenus, Myzorynchus Series), Annularis, Jamesii, Maculatus ( *Cellia* Subgenus, Neocellia Series), Funestus ( *Cellia* Subgenus, Myzomyia Series), Kochi, Leucosphyrus, Tessellatus ( *Cellia* Subgenus, Neomyzomyia Series) and Subpictus ( *Cellia* Subgenus, Pyretophorus Series). *Anopheles karwari* ( *Cellia* Subgenus, Neocellia Series) was also detected at a low frequency. Less than 5% (6010/129228) of the specimens could not be identified at the Group level because they were damaged (missing legs or wings).

**Figure 2.**
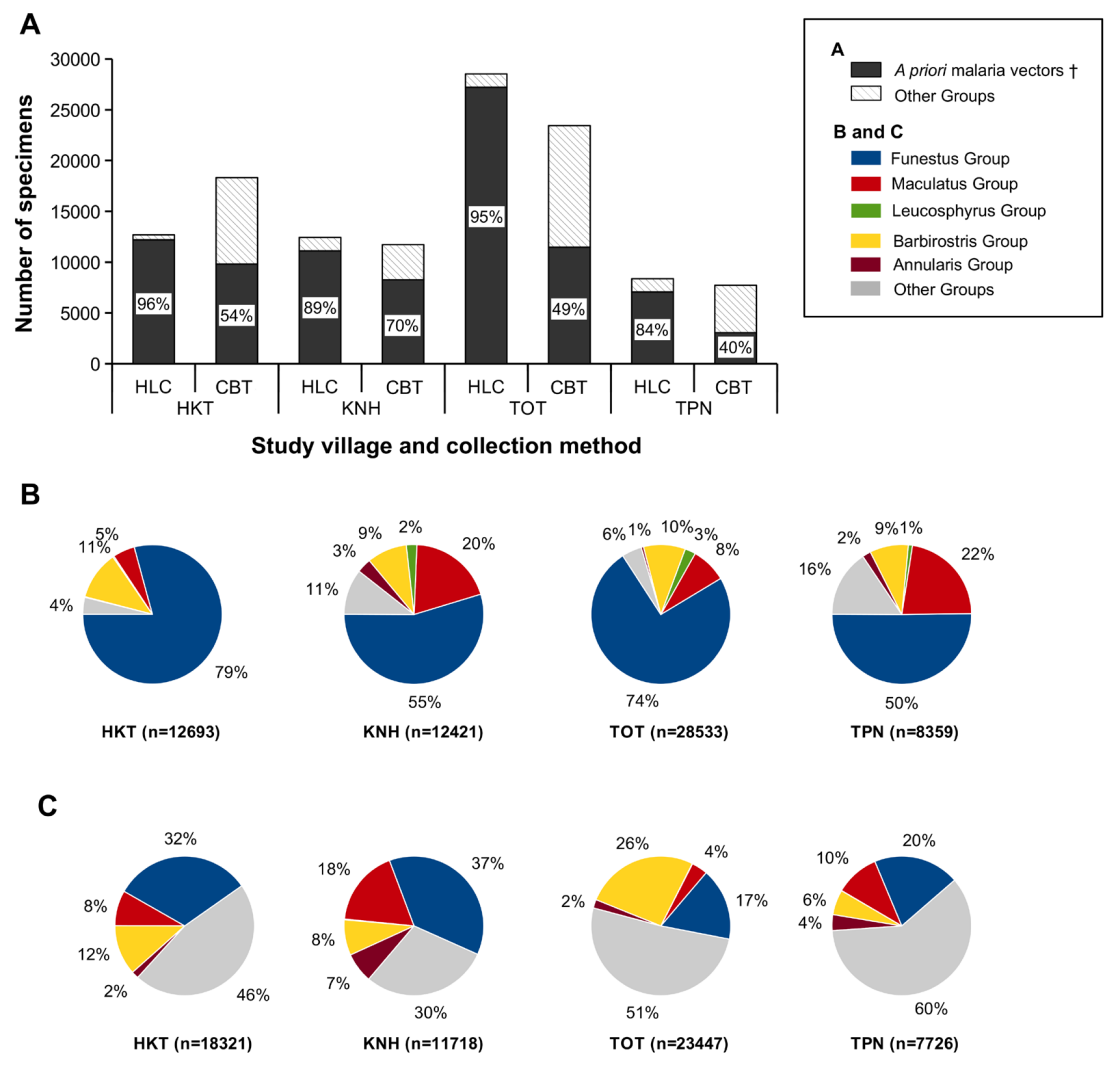
Biodiversity of the *Anopheles* mosquitoes according to the collection method and study village. **A**) Proportion of malaria vectors in *Anopheles* populations collected by human-landing catch (HLC) and cow bait trap (CBT) collection methods according to the village. **B**) Relative proportions of *sensu lato* malaria vectors collected by HLC according to the village. **C**) Relative proportions *sensu lato* malaria vectors collected by CBT according to the village. † *A priori* malaria vectors refers to *Anopheles* taxa that were previously reported to be infected with human malaria parasites in the Thailand-Myanmar border area, *i.e.* the Funestus, Maculatus, Leucosphyrus, Barbirostris and Annularis Groups ^11– 17^.

*A priori* malaria vectors in the Annularis, Barbirostris, Funestus, Leucosphyrus and Maculatus Groups ( *i.e. Anopheles* taxa that were previously reported to be infected with human malaria parasites in the Thailand-Myanmar border area) accounted for 84–96% and for 40–70% of the *Anopheles* mosquitoes collected by HLC and CBT collection methods respectively ( Figure 2). The abundance of malaria vectors was significantly different from one collection site to another in a given village, suggesting that local ecological factors are important drivers of exposure to malaria vectors ( Supplementary File 1, Figures S1–S4). The proportion of collection-night during which a given *Anopheles* taxa was collected varied between 3% and 75% in the Subpictus and Funestus Groups respectively ( Supplementary File 1, Figures S5–S6). The Funestus Group was the most abundant taxa during both the rainy and dry seasons (June to November and December to May, respectively). The Maculatus and Leucosphyrus Groups were mainly collected during the rainy season. The abundance of Annularis and Barbirostris Groups peaked during the transition period between the rainy and dry seasons, when the abundance of other groups decreased ( Supplementary File 1, Figures S7–S10).

*Anopheles minimus* ( *s.s.*) was by far the most abundant species among the Funestus Group ( Table 2). Its sibling *An. harrisoni* and closely related members from the Aconitus and Culicifacies Subgroups represented <0.5% and 13% of the specimens from the Funestus populations collected by HLC and CBT, respectively. Aconitus and Culicifacies Subgroups represented up to 12% of the Funestus specimens in TPN ( Supplementary File 1, Table S2). *Anopheles maculatus* ( *s.s.*), *An. sawadwongporni* and *An. pseudowillmori* were the most abundant species in the Maculatus Group with proportion varying from 15 to 50%, 40 to 81% and 3.6 to 9% according to the village. Three species from the Dirus Complex accounted for >99% of the specimens in the Leucosphyrus Group, namely *An. dirus* ( *s.s.*) *An baimaii* and *An. cracens*. The proportion of *An. dirus* ( *s.s.*) and *An baimaii* in study villages varied from 60 to 97% and from 0 to 39%, respectively. The proportion of *An. culicifacies* B, *An. maculatus* ( *s.s.*) and *An. cracens* increased during the dry season whereas that of *An. minimus* ( *s.s.*), *An. pseudowillmori* and *An. baimaii* increased during the rainy season ( Supplementary File 1, Table S3).

**Table 2.** Molecular identification of sibling species among the Funestus, Maculatus and Leucosphyrus Groups.

Group	Collection method (% of collected specimen analyzed by PCR)	Species	n/N	Relative proportion estimate	95%CI
Funestus	HLC (3294/42283=8%)	*An. minimus* ( *s.s.*)	3277/3294	99.5	99.2-99.7
		*An. aconitus* ( *s.s.*)	7/3294	0.2	0.1-0.4
		*An. culicifacies B*	6/3294	0.2	0.1-0.4
		*An. harrisoni*	2/3294	0.1	0-0.2
		*An. varuna*	2/3294	0.1	0-0.2
		*An. pampanai*	0/3294	0	-
	CBT (1543/15728=10%)	*An. minimus* ( *s.s.*)	1342/1543	87	85.2-88.6
		*An. culicifacies B*	90/1543	5.8	4.7-7.1
		*An. varuna*	59/1543	3.8	2.9-4.9
		*An. aconitus* ( *s.s.*)	42/1543	2.7	2-3.7
		*An. pampanai*	9/1543	0.6	0.3-1.1
		*An. harrisoni*	1/1543	0.1	0-0.4
Maculatus	HLC (1476/7281=20%)	*An. sawadwongporni*	819/1476	55.5	52.9-58
		*An. maculatus* ( *s.s.*)	537/1476	36.4	33.9-38.9
		*An. pseudowillmori*	114/1476	7.7	6.4-9.2
		*An. dravidicus*	6/1476	0.4	0.1-0.9
	CBT (1491/5239=28%)	*An. sawadwongporni*	975/1491	65.4	62.9-67.8
		*An. maculatus* ( *s.s.*)	439/1491	29.4	27.1-31.8
		*An. pseudowillmori*	74/1491	5	3.9-6.2
		*An. dravidicus*	3/1491	0.2	0-0.6
Leucosphyrus	HLC (856/1144=77%)	*An. baimaii*	643/856	75.1	72.1-78
		*An. dirus* ( *s.s.*)	205/856	23.9	21.1-27
		*An. cracens*	5/856	0.6	0.2-1.4
		*An. balabacensis*	3/856	0.4	0.1-1
	CBT (29/46=57%)	*An. dirus* ( *s.s.*)	14/29	48.3	29.4-67.5
		*An. baimaii*	10/29	34.5	17.9-54.3
		*An. cracens*	5/29	17.2	5.8-35.8
		*An. balabacensis*	0/29	0	-

### Malaria vectors

The contribution of six Groups of *Anopheles* to malaria transmission was determined by processing 56872 samples collected by HLC in qPCR *Plasmodium*. Human malaria parasites *P. falciparum* and *P. vivax* were detected in 106 *Anopheles* specimens that belonged to four Groups of *Anopheles* species (Funestus, Maculatus, Leucosphyrus and Barbirostris) ( Supplementary File 1, Table S4). Both *P. falciparum* and *P. vivax* were detected in the Funestus, Leucosphyrus and Maculatus Groups, whereas only *P. vivax* was detected in the Barbirostris Group. One specimen of the Funestus Group was co-infected with both *P. falciparum* and *P. vivax*. The Funestus Group was by far the most important taxa contributing to malaria transmission (Pf-EIR= 0.1 and Pv-EIR= 0.6 infective bites/person/month) followed by the Maculatus, Leucosphyrus and Barbirostris Groups ( Table 3). Due to the relatively low sample size analyzed in the Annularis and Subpictus Groups (747 and 126 respectively), it was not possible to rule out their contribution to malaria transmission.

**Table 3.** Entomological indices of malaria transmission presented per Group of *Anopheles*.

Group	HBR (b/p/m)			Pf-SI (%)			Pv-SI (%)			Pf-EIR (ib/p/m)			Pv-EIR (ib/p/m)		
	n/N	Estimate	95%CI	n/N	Estimate	95%CI	n/N	Estimate	95%CI	n/N	Estimate	95%CI	n/N	Estimate	95%CI
Funestus	42283/4120	307.9	305-310.8	13/41797	0.03	0.02-0.05	77/41797	0.18	0.15-0.05	13/4073	0.1	0.05-0.16	77/4073	0.57	0.45-0.71
Maculatus	7281/4120	53	51.8-54.2	4/7178	0.06	0.02-0.14	7/7178	0.1	0.04-0.14	4/4062	0.03	0.01-0.08	7/4062	0.05	0.02-0.11
Leucosphyrus	1144/4120	8.3	7.9-8.8	1/1107	0.09	0-0.5	3/1107	0.27	0.06-0.5	1/3987	0.01	0-0.04	3/3987	0.02	0-0.07
Barbirostris	6098/4120	44.4	43.3-45.5	0/5917	0	0-0.06	2/5917	0.03	0-0.06	0/3998	0	0-0.03	2/3998	0.02	0-0.05
Annularis	772/4120	5.6	5.2-6	0/747	0	0-0.49	0/747	0	0-0.49	0/3987	0	0-0.03	0/3987	0	0-0.03
Subpictus	144/4120	1	0.9-1.2	0/126	0	0-2.89	0/126	0	0-2.89	0/3605	0	0-0.03	0/3605	0	0-0.03

**b/p/m**: bites /person /month; **CI**: confidence interval; **HBR**: human-biting rate; **ib/p/m**: infective bites /person /month; **n/N**: value of the numerator and denominator in the calculation of the corresponding indice as per the definition given in the Methods section; **Pf-EIR**: *P. falciparum* entomological inoculation rate; **Pv-EIR**: *P. vivax* entomological inoculation rate; **Pf-SI**: *P. falciparum* sporozoite index; **Pv-SI**: *P. vivax* sporozoite index.

*Plasmodium*-infected mosquitoes were identified at the species level using molecular assays. Six formally named species were incriminated in malaria transmission: *An. minimus* ( *s.s.*), *An. aconitus* ( *s.s.*) (Funestus Group), *An. maculatus* ( *s.s.*), *An. sawadwongporni* (Maculatus Group), *An. dirus* ( *s.s.*) and *An. baimaii* (Leucosphyrus Group). *Plasmodium vivax* sporozoites were detected in all species whereas *P. falciparum* sporozoites were detected only in *An. minimus* ( *s.s.*), *An. maculatus* ( *s.s.*), *An. sawadwongporni* and *An. dirus* ( *s.s.*). Molecular identification at the species level was not possible for 6/106 positive samples because there was no DNA remaining.

Interestingly, the avian malaria *P. juxtanucleare* was detected in five specimens of the Funestus, Maculatus and Leucosphyrus Groups collected by HLC (two *An. minimus* ( *s.s.*), one *An. baimaii* and two undetermined species). In addition, 16% (3308/21013) of the specimens from the Funestus, Maculatus and Leucosphyrus Groups collected in the CBTs were screened for *Plasmodium* sporozoites. *Plasmodium vivax* sporozoites were detected in two *An. minimus* ( *s.s.*).

**Figure 3.**
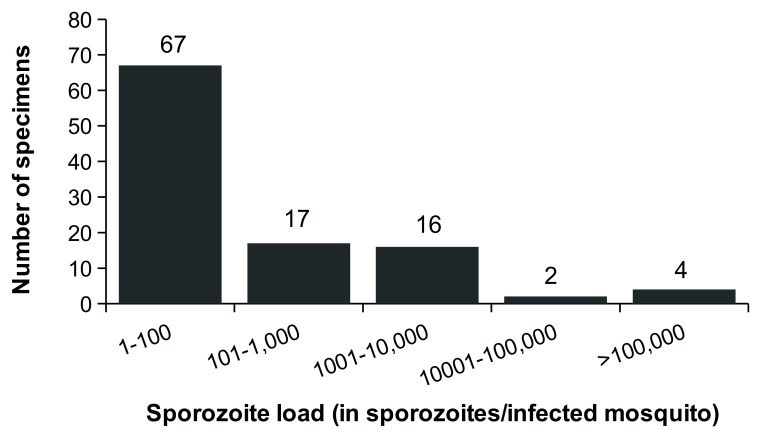
Frequency distribution of the sporozoite load in naturally infected malaria vectors.

Quantitation of the sporozoite load was possible in 106/108 of the *P. falciparum* and *P. vivax* positive samples. Overall, 63% (67/106) of the infected specimens carried less than 100 sporozoites ( Figure 3). The geometric mean was 41 (95%CI= [14; 98]) and 162 (95%CI= [94; 167]) sporozoites /infected mosquito for *P. falciparum* and *P. vivax* respectively ( Supplementary File 1, Table S5). *Anopheles maculatus* ( *s.l.*) appeared to be infected with lower parasite loads compared to other anopheline species. The range of sporozoite loads was 6 to 9234 sporozoites for *P. falciparum* and 4 to 517500 sporozoites for *P. vivax*. Moreover, 81/108 abdomens from sporozoite-positive samples were screened for *Plasmodium* oocysts (remaining abdomens were lost or moldy). *Plasmodium* oocysts were detected in only 57% (46/81) of the sporozoites-positive specimens. Thirty-two out of the 35 sporozoites-positive oocysts-negative specimens carried less than 500 sporozoites in their salivary glands, suggesting that these specimens were infected with a low number of oocysts.

### Blood-seeking behaviour of *Anopheles* mosquitoes

Taking into account the whole dataset, the mean HBR of *Anopheles* mosquitoes was 460 bites/person/month (95%CI= [457; 463]) and the mean CBR was 4807 bites/cow/month (95%CI= [4770; 4843]). Mean HBR of primary malaria vectors varied from 2 to 306 bites/person/month in *An. dirus* ( *s.s.*) and *An. minimus* ( *s.s.*) respectively. Some primary malaria vectors were also frequently collected on CBT: *Anopheles minimus* ( *s.s.*), *An. sawadwongporni* and *An. maculatus* ( *s.s.*) had a mean CBR of 996, 249 and 112 bites/cow/month respectively ( Supplementary File 1, Figure S11). The data on secondary vectors ( *e.g.* Barbirostris and Annularis Groups) are more difficult to interpret because they probably represent a mix of several sibling species.

**Figure 4.**
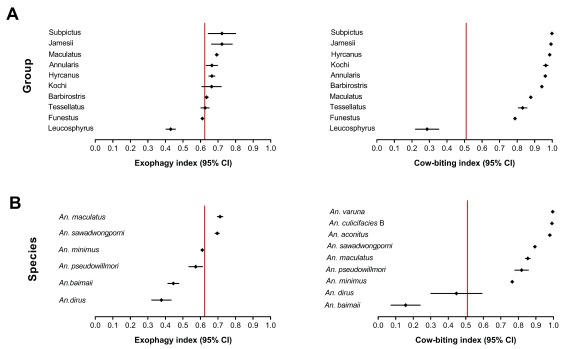
Exophagy index (EI) and zoophagy index (CBI) of *Anopheles* mosquitoes. **A**) EI and CBI are presented at the Group level. **B**) EI and CBI are presented at the species level. Vertical red lines show the mean value of the indice for the *Anopheles spp.* mosquitoes.

Leucosphyrus members were the most anthropophagic and endophagic species (mean CBI=0.16 and 0.44 and mean EI= 0.45 and 0.37 in *An. baimaii* and *An. dirus* ( *s.s.*) respectively). Other malaria vectors in the Funestus, Maculatus and Barbirostris Groups were more zoophagic and exophagic (mean CBI=0.75-0.95 and mean EI=0.60-0.75). All remaining species were strongly zoophagic and exophagic (mean CBI= 0.83-1.00 and mean EI= 0.63-0.83) ( Figure 4). Beyond zoophagy, malaria vectors appeared opportunistic in the choice of their blood meal source. Indeed, we have shown that *Anopheles* specimens collected by HLC can carry the avian malaria parasite *P. juxtanucleare*, and that *Anopheles* specimens collected on CBT can carry the human malaria parasite *P. vivax* ( *i.e. Anopheles* mosquitoes collected on a given host type can carry *Plasmodium* sporozoites acquired from a previous blood meal taken on a different host type). These findings clearly demonstrate that malaria vectors can feed alternatively on different blood sources during their life span.

**Figure 5.**
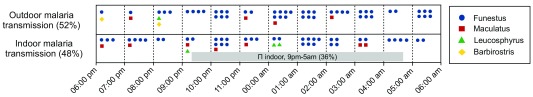
Distribution of infective bites according to the collection time and location. π _indoors, 9pm-5am_: proportion of the *Plasmodium*-infected mosquitoes collected indoors between 09:00 pm and 05:00 am.

*Anopheles* mosquitoes exhibited an outdoor and/or early biting pattern ( Supplementary File 1, Figures S12 and S13). Some species were already active at 06:00 pm and/or at 06:00 am, suggesting that exposure to malaria vectors stretches out of the standard collection time. The proportion of malaria vectors (both *Plasmodium*-infected and uninfected specimens) collected indoors between 09:00 pm and 05:00 am was 29% (range= 15–48% according to the species). Conversely, 64% of the *Plasmodium*-infected specimens were collected either out of doors, or indoors before 09:00 pm and after 05:00 am ( Figure 5).

### Entomological indices of malaria transmission

Only the Funestus, Maculatus and Leucosphyrus Groups were taken into account for the analysis of the entomological indices. Overall, mean HBR was 369 bites/person/month (95%CI= [366; 372]) and compensated for the low infection rate in these naturally infected populations of malaria vectors. Mean Pf-SI was 0.04% (95%CI= [0.02; 0.06]) and mean Pv-SI was 0.17% (95%CI= [0.14; 0.21]), yielding mean Pf-EIR of 0.13 (95%CI= [0.08; 0.21]) and mean Pv-EIR of 0.64 (95%CI= [0.51; 0.79]) infective bites/person/month ( Table 4). The transmission of *P. falciparum* was highly seasonal: the rainy season was associated with a 10-fold increase in Pf-EIR. In contrast, mean Pv-EIR was 0.52 (95%CI= [0.34; 0.75]) and 0.73 (95%CI= [0.55; 0.94]) infective bites/person/month during the rainy season and the dry season respectively ( Table 5). Only 6% (1/18) of the mosquitoes infected with *P. falciparum* sporozoites were detected during the dry season whereas 32% (28/87) of the mosquitoes infected with *P. vivax* sporozoites were detected during the dry season (two-sided Fisher’s exact test, p-value= 0.0218).

**Table 4.** Entomological indices presented per study village.

Village	HBR (bites/person/month)			Pf-SI (%)			Pv-SI (%)			Pf-EIR (infective bites/person/month)			Pv-EIR (infective bites/person/month)		
	n/N	Estimate	95%CI	n/N	Estimate	95%CI	n/N	Estimate	95%CI	n/N	Estimate	95%CI	n/N	Estimate	95%CI
HKT	10736/1020	316	310-322	5/10625	0.05	0.02-0.11	39/10625	0.37	0.26-0.5	5/1009.5	0.15	0.05-0.35	39/1009.5	1.16	0.82-1.58
KNH	9536/1050	272	267-278	4/9414	0.04	0.01-0.11	27/9414	0.29	0.19-0.42	4/1036.6	0.12	0.03-0.3	27/1036.6	0.78	0.51-1.14
TOT	24293/1050	694	685-703	6/24083	0.02	0.01-0.05	15/24083	0.06	0.03-0.1	6/1040.9	0.17	0.06-0.38	15/1040.9	0.43	0.24-0.71
TPN	6143/1000	184	180-189	3/5960	0.05	0.01-0.15	6/5960	0.1	0.04-0.22	3/970.2	0.09	0.02-0.27	6/970.2	0.19	0.07-0.4
All villages	50708/4120	369	366-372	18/50082	0.04	0.02-0.06	87/50082	0.17	0.14-0.21	18/4069.1	0.13	0.08-0.21	87/4069.1	0.64	0.51-0.79

**b/p/m**: bites /person /month; **CI**: confidence interval; **HBR**: human-biting rate; **ib/p/m**: infective bites /person /month; **n/N**: value of the numerator and denominator in the calculation of the corresponding indice as per the definition given in the Methods section; **Pf-EIR**: *P. falciparum* entomological inoculation rate; **Pv-EIR**: *P. vivax* entomological inoculation rate; **Pf-SI**: *P. falciparum* sporozoite index; **Pv-SI**: *P. vivax* sporozoite index.

**Table 5.** Entomological indices presented per season.

Season	HBR (bites/person/month)			Pf-SI (%)			Pv-SI (%)			Pf-EIR (infective bites/person/month)			Pv-EIR (infective bites/person/month)		
	n/N	Estimate	95%CI	n/N	Estimate	95%CI	n/N	Estimate	95%CI	n/N	Estimate	95%CI	n/N	Estimate	95%CI
Dry ^a^	39259/2470	477	472-482	17/38777	0.04	0.03-0.07	59/38777	0.15	0.12-0.2	17/2439.7	0.21	0.12-0.33	59/2439.7	0.73	0.55-0.94
Rainy ^b^	11449/1650	208	204-212	1/11305	0.01	0-0.05	28/11305	0.25	0.16-0.36	1/1629.2	0.02	0-0.1	28/1629.2	0.52	0.34-0.75
All seasons	50708/4120	369	366-372	18/50082	0.04	0.02-0.06	87/50082	0.17	0.14-0.21	18/4069.1	0.13	0.08-0.21	87/4069.1	0.64	0.51-0.79

^a^ December to May ^b^ June to December **b/p/m**: bites /person /month; **CI**: confidence interval; **HBR**: human-biting rate; **ib/p/m:** infective bites /person /month; **n/N**: value of the numerator and denominator in the calculation of the corresponding indice as per the definition given in the Methods section; **Pf-EIR**: *P. falciparum* entomological inoculation rate; **Pv-EIR**: *P. vivax* entomological inoculation rate; **Pf-SI**: *P. falciparum* sporozoite index; **Pv-SI**: *P. vivax* sporozoite index.

**Figure 6.**
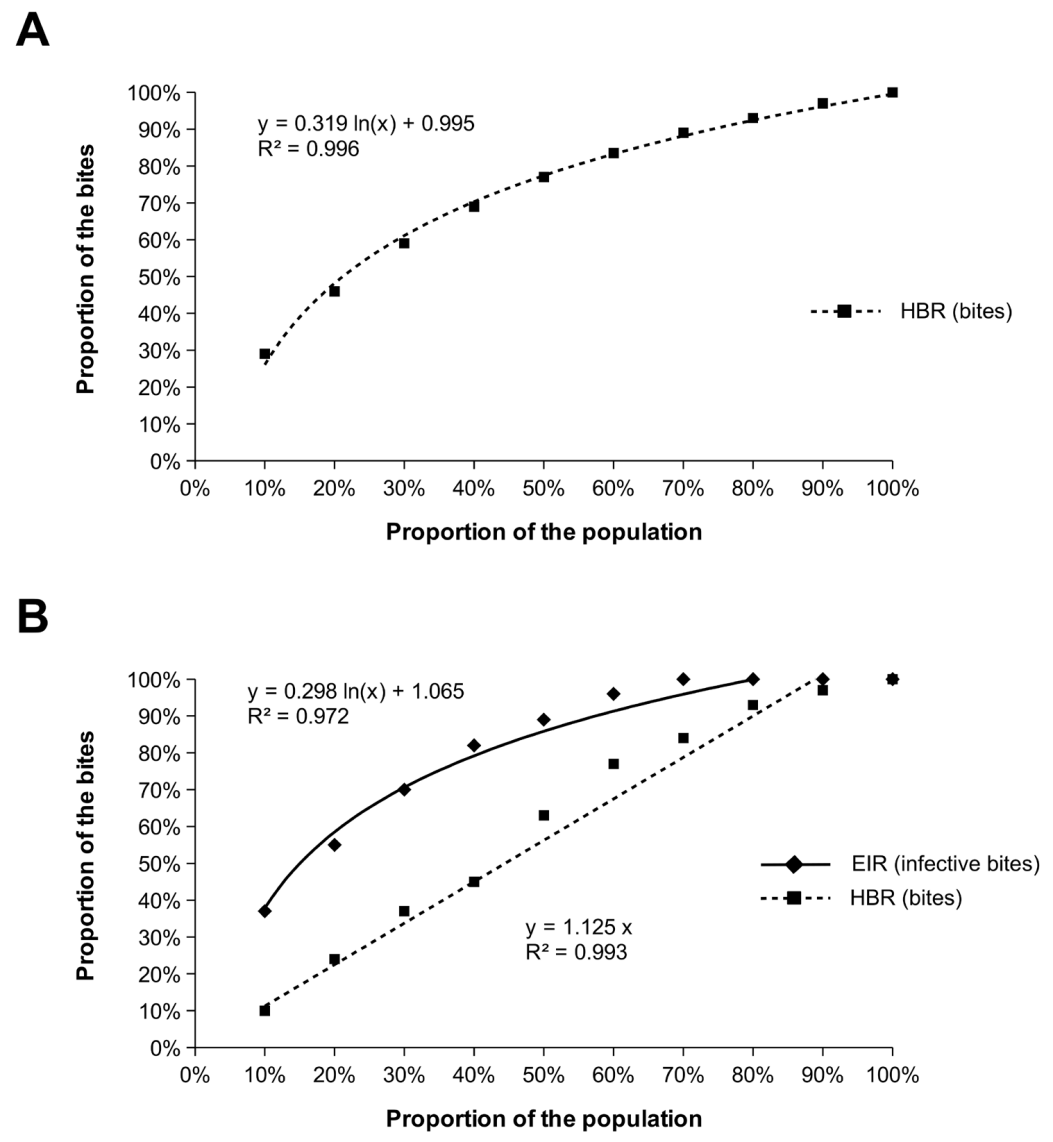
Heterogeneous distribution of bites and infective bites among the cohort of mosquito collectors over the entire period of the study. **A**) Cumulative distribution of the bites of malaria vectors among the cohort of mosquito collectors. **B**) Paired cumulative distribution of bites of malaria vectors and infective bites among the cohort of mosquito collectors.

Average values of entomological indices concealed a high heterogeneity. When data were aggregated at the village and survey levels, mean HBR ranged from 13 to 2611 bites/person/month, mean Pf-EIR ranged from 0.00 to 3.05 infective bites/person/month and mean Pv-EIR ranged from 0.00 to 9.75 infective bites/person/month ( Supplementary File 1, Figure S7–S10). The lowest HBR measured on a single collector and during a single night of collection was 0 bites/night and the highest was 289 bites/night. When taking into account the entire follow-up, mean HBR measured on single collectors ranged from 66 to 1253 bites/person/month, mean Pf-EIR ranged from 0 to 0.86 infective bites/person/month and mean Pv-EIR ranged from 0 to 4.92 infective bites/person/month (100–105 collection nights/mosquito collector). The cumulative HBR and EIR measured in the cohort of mosquito collectors followed a logarithmic distribution: 20% of the collectors received 50% of the bites and 20% of the collectors received 50% of the infective bites. In contrast, 30% of the collectors did not receive any infective bites during the study. Interestingly, the cumulative HBR followed a linear trend when paired to EIR, suggesting that the heterogeneity in the distribution of infective bites was not explained by the mean of exposure to malaria vectors ( Figure 6).

## Discussion

This study was a unique opportunity to document some entomological aspects of malaria transmission in low transmission settings of Southeast Asia. Our data are important in the context of malaria elimination locally in Kayin state, but also elsewhere in the Greater Mekong Subregion where malaria displays a similar transmission pattern.

### The dynamics of entomological indices in an area of low, seasonal and unstable *P. falciparum* transmission setting

Our results confirm previous observations that primary malaria vectors in this area are *An. minimus* ( *s.s.*), *An. maculatus* ( *s.s.*), *An. sawadwongporni*, *An. dirus* ( *s.s.*) and *An. baimaii*, and that several other species also play a secondary role in the transmission. Infection rate is low in naturally infected populations of malaria vectors and balanced by the high biting-rate of malaria vectors ^11, 15^, yielding mean entomological inoculation rate of 1.6 and 7.7 infective bites/person/year for *P. falciparum* and *P. vivax* respectively. These EIR estimates were made in the context of falciparum malaria elimination (community wide access to early diagnosis and treatment, and mass drug administration). Therefore, baseline intensity of falciparum malaria transmission in hotspot villages in Kayin state is likely to be higher than that reported in this study ^15^. In contrast, *Plasmodium vivax* EIR estimates were higher than previously reported in the same area ^11^. This implies that new infections (primo-infections and reinfections) are more common than previously thought and probably frequently asymptomatic ^32^.

The transmission of *P. falciparum* was seasonal whereas infective bites of *P. vivax* occurred during both the dry and rainy seasons. The seasonality in *P. falciparum* transmission was only partially explained by the increase in malaria vector abundance during the rainy season when compared to the dry season. The longevity of malaria vectors was most likely to be the main factor driving the seasonality of *P. falciparum* transmission. During the dry season, the life expectancy of malaria vectors was probably too short for *P. falciparum* to complete its sporogonic cycle in the mosquito. During the rainy season, the longevity of malaria vectors may have increased and malaria vectors lived long enough for *P. falciparum* sporozoites to appear in the salivary glands ^33, 34^. For *P. vivax*, the duration of the sporogonic cycle was compatible with sporozoite detection throughout the year as this parasite develops faster than any other species in its mosquito vectors ^35, 36^. Relapsing vivax malaria parasites, vector competency and temperature are also potential factors that explain seasonal trends in malaria transmission.

Although 5% of the mosquitoes infected with *Plasmodium* sporozoites had >10,000 sporozoites in their salivary glands, the sporozoite load measured in these naturally infected malaria vectors was in average very low (60% of the infected specimens carried less than 100 sporozoites). This is consistent with previous attempts to quantify *P. falciparum* and *P. vivax* sporozoites in low transmission settings ^24, 37– 39^ and contrasts with the high sporozoite loads detected in Africa ^40– 42^. Considering a geometric mean of 162 sporozoites / *P. vivax* infected mosquito, a mean *P. vivax* EIR of 7.7 infective bites /person /year and that infected mosquitoes would transmit all their sporozoites, we estimated that each person would receive 1200 *P. vivax* sporozoites per year in average. Taking into account that not all sporozoites may be inoculated and that not all sporozoites may transform into hypnozoites, the reservoir of hypnozoite is likely to be small in most individuals exposed to the disease in this transmission setting.

The distribution of infective bites among the human population was highly heterogeneous. This pattern was not explained by the mean of exposure to malaria vectors as paired cumulative HBR and EIR did not follow the same trend. The study villages were hotspots of malaria transmission defined by the high prevalence of asymptomatic infection ^19^. This implies a substantial degree of premunition in asymptomatic carriers, *i.e.* the development of a protective immunity that maintains parasitaemia at sub-patent levels. The broad spectrum of EIRs measured in hotspot villages may explain why some people develop such a protective immunity and manage to control the infection.

### Residual malaria transmission

The two broadly scalable vector-control interventions recommended by the World Health Organization for the control of malaria vectors are mass distribution of LLINs or, where appropriate, IRS campaigns ^43^. By definition, LLINs target malaria vectors seeking human blood, indoors and at a time when people are sleeping under mosquito nets. In order IRS to be effective, malaria vectors targeted by the intervention must also rest indoors, before or after a blood meal. However, this stereotypical blood seeking behavior applies only to a minority of the dominant malaria vectors worldwide ^44^. Several behavioral traits drive the refractoriness of malaria vectors to LLINs and IRS including *(i)* their ability to take blood meals from animals (zoophagy and opportunistic blood type selection), *(ii)* their tendency to rest and/or feed outdoors (exophily and exophagy) and *(iii)* their ability to feed before dawn and after dusk, at a time when people are not protected by LLINs or IRS intervention ^9^.

As previously reported, LLINs only have a limited efficacy in preventing human-vector contact and disease transmission in the Thailand-Myanmar border area. Somboon *et al*. evaluated the impact of mosquito bed nets impregnated with lambda-cyhalothrin using entomological endpoints in very similar transmission settings (Karen villages located on the Thai side of the border) ^7^. The authors reported that mosquito bed nets can prevent 36–78% of the human-vector contact according to the *Anopheles* species. Universal coverage with LLINs failed to reduce the abundance and longevity of malaria vectors, suggesting that this intervention had only a limited impact on the vectorial capacity. The impact of permethrin-impregnated mosquito bed nets was also evaluated in pregnant women and children living in refugee camps using epidemiological endpoints. The use of mosquito bed nets during pregnancy was associated with a significant reduction in the incidence of severe anaemia but not of malaria ^45^. At a time when EIR was higher, the use of mosquito bed nets in children was associated with a significant decrease of falciparum malaria incidence but no effect was observed on *P. vivax* ^46^. More recently, Smithuis *et al*. failed to observe an impact of LLINs among a cohort of 175 children followed for 10 months in Western Myanmar ^47^. This negative result was explained by the early and outdoor biting pattern of malaria vectors ^48^.

In this study, only 36% of the *Plasmodium*-infected mosquitoes were collected indoors between 09:00 pm and 05:00 am (when and where people are expected to sleep under a bed-net). This proportion might have been overestimated as malaria vectors were already active at 06:00 pm and/or at 06:00 am, suggesting that the exposure stretched out of the collection time. Accurate quantitation of the part of the transmission that LLINs fail to prevent would require collecting additional data on population movements and sleeping habits of people living in this area. Moreover, we have clearly demonstrated an opportunistic blood type selection in some vectors, *i.e.* that a given specimen is able to feed on several blood sources during successive gonotrophic cycles. This opportunism also appears as an important factor to explain why universal coverage with mosquito bed nets fails to affect the dynamic of anopheline populations and decrease vectorial capacity in the area ^7^. Consequently, the paradigm of residual transmission as experienced in high transmission settings of Africa does not apply to the Thailand-Myanmar border area and a drastic shift in vector-control interventions is required.

### Shift in vector-control intervention

The design of effective intervention for the control of malaria vectors in Southeast Asia should take into account malaria vector ecology and transmission dynamics. In this study, we have shown that multiple vectors have different and complementary blood-seeking behaviours, making their control particularly difficult.

Veterinary approaches such as the injection of livestock with a slow-release formulation of endectocides ^49^, or the use of insecticide-treated mosquito nets fenced around cattle ^50^ may be an interesting strategy to decrease the vectorial capacity of some zoophilic and/or zoophagic malaria vectors (ex: *An. minimus*, *An. maculatus* and *An. sawadwongporni*). We have shown that malaria vectors can readily feed on a wide variety of blood types including human, cattle, pigs and birds. However, the diversity of blood sources and the relative proportion of blood meals taken on a given source remain to determine. In this regard, targeted sequencing of mammalian mt 16S ribosomal RNA genes detected in DNA extracts from blood-fed specimens is a promising tool for the determination of blood-meal sources in wild populations of malaria vectors ^51^.

The nature of *Anopheles* resting habitats is another important aspect of malaria vector ecology that can be targeted by residual insecticide spraying intervention. Resting habitats have been identified both indoors (ex: roof, wall, ceilings of houses and animal barns) and outdoors (ex: tree holes, rodent holes, dense bushes) ^52^. However, most mosquito species rest exclusively out of doors in natural settings, and only a relatively few species rest in man-made shelters ^52^. The size and importance of the exophilic population that commonly rest inside houses are typically overlooked because the sampling of outdoor-resting population is more difficult than the sampling of indoor-resting population. In Southeast Asia, most of the life cycle of *Anopheles* mosquitoes is likely to take place out of doors and malaria vectors are barely never found resting indoors ^18^. Therefore, the scope of residual insecticide spraying for the control of malaria vectors may be extended to outdoor applications.

Insecticide resistance may also represent an additional threat to the control of malaria vectors in this area. We have previously detected relatively low levels of resistances to deltamethrin and permethrin in populations of *An. minimus* ( *s.l.*) and *An. maculatus* ( *s.l.*) collected in the villages of the present study ^53^. Further investigations are needed in order to document the extent of pyrethroid resistance elsewhere in Kayin state and to evaluate the potential effectiveness of alternative class of insecticides such as carbamate (ex. bendiocarb), organophosphate (ex. malathion) or insect growth inhibitors (ex. pyriproxyfen).

## Conclusion

This study highlights the importance of entomology in the context of malaria elimination in Kayin state. A drastic shift in vector-control strategy is required in order to address early and outdoor malaria transmission, and the modalities of vector-control should be retuned to address problematic specific to malaria elimination.

## Data availability

The data is available upon request to the Mahidol Oxford Tropical Medicine Research Unit Data Access Committee ( Supplementary File 3; http://www.tropmedres.ac/data-sharing) and following the Mahidol Oxford Tropical Medicine Research Unit data access policy ( http://www.tropmedres.ac/_asset/file/data-sharing-policy-v1-0.pdf).

## Supplementary material

**Supplementary File 1.** Supplemental data.

**Supplementary File 2. Laboratory procedures for the processing of entomological samples.**

**Supplementary File 3.** Request form for Mahidol Oxford Tropical Medicine Research Unit Data Access Committee.
