# Supplementary material for: Entomological determinants of malaria transmission in Kayin state, Eastern Myanmar: A 24-month longitudinal study in four villages
Source: Wellcome Open Res. 2019 Jun 17;3:109. Originally published 2018 Aug 31. [Version 4] doi: 10.12688/wellcomeopenres.14761.4 (PMC6544137; doi:10.12688/wellcomeopenres.14761.4)
Supplement: Supplementary file 1 [file wellcomeopenres-3-16720-s0000.tgz › b824ae03-5cf8-4e16-ab68-5f27a7d9f8e7_Supp_file_1_revised.docx]

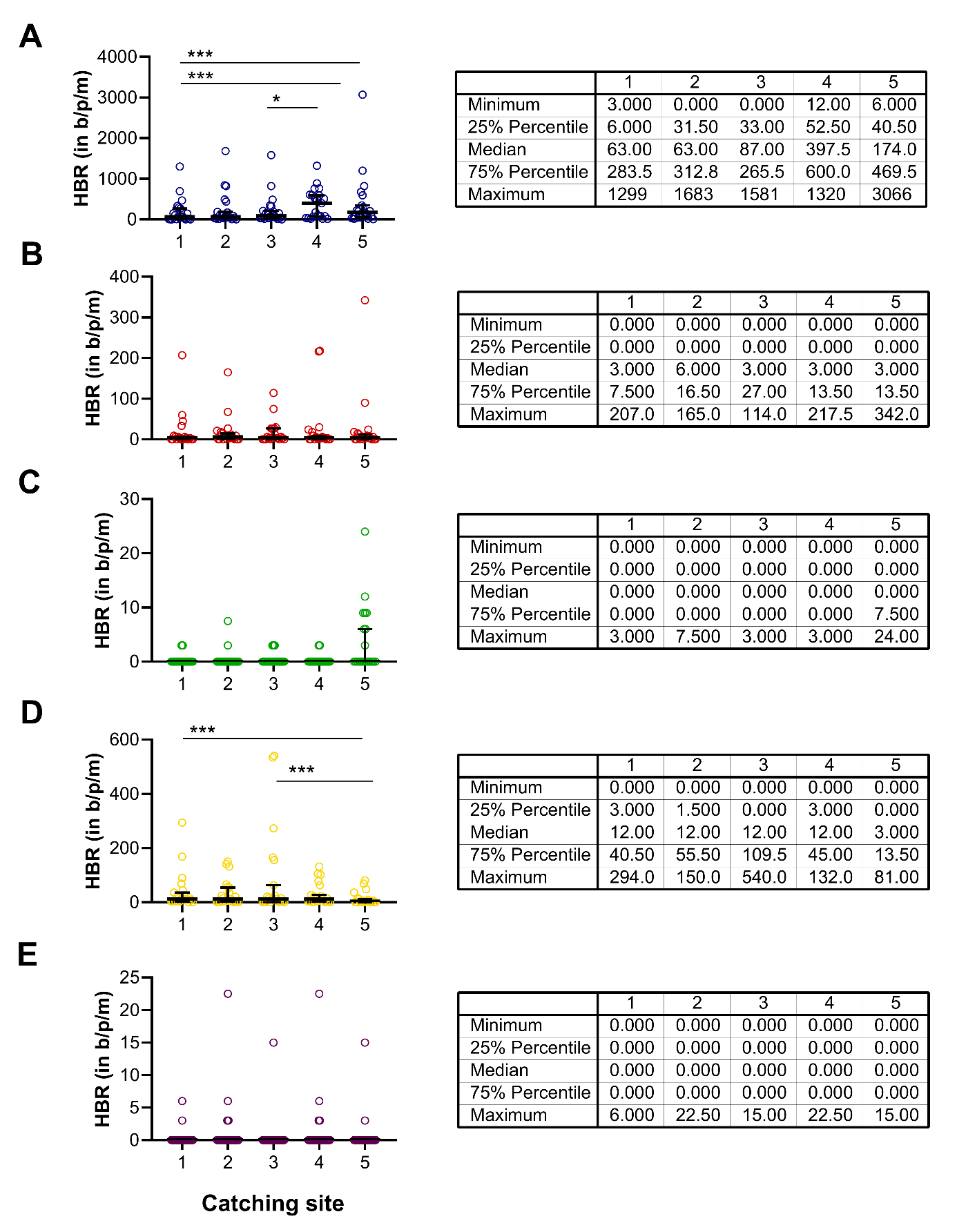


**Figure S1. Spatial heterogeneity in malaria vectors distribution in HKT.** A) Funestus Group, B) Maculatus Group, C) Leucosphyrus Group, D) Barbirostris Group, E) Annularis Group. Human-biting rate (HBR) estimates were collated per catching site for each entomological survey. The statistical significance of the differences observed between catching sites was assessed with the Friedman test and a post-hoc analysis. Dunn’s correction was applied for multiple comparisons. *p* <0.033 (*), <0.002 (**) and <0.001 (***).


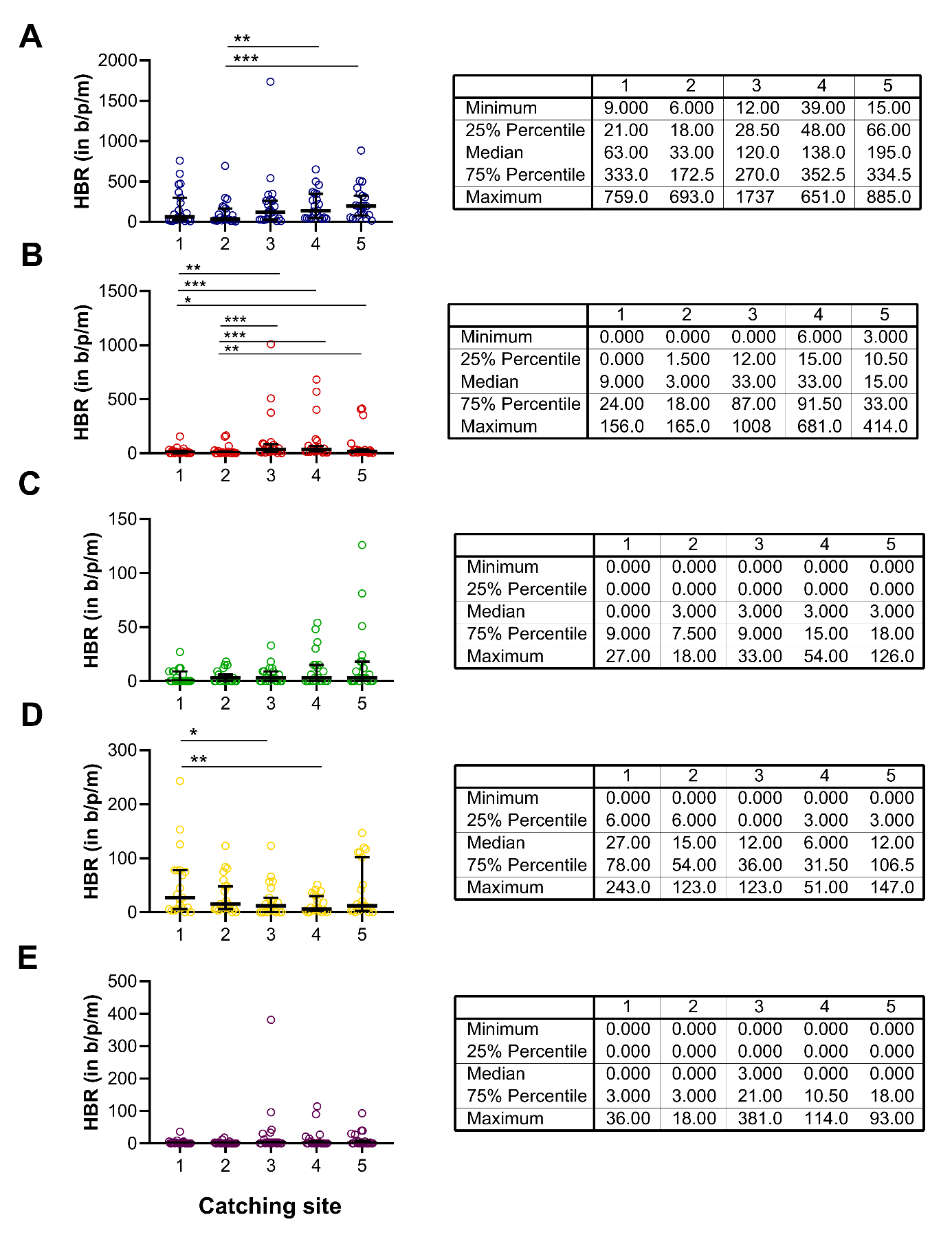


**Figure S2. Spatial heterogeneity in malaria vectors distribution in KNH.** A) Funestus Group, B) Maculatus Group, C) Leucosphyrus Group, D) Barbirostris Group, E) Annularis Group. Human-biting rate (HBR) estimates were collated per catching site for each entomological survey. The statistical significance of the differences observed between catching sites was assessed with the Friedman test and a post-hoc analysis. Dunn’s correction was applied for multiple comparisons. *p* <0.033 (*), <0.002 (**) and <0.001 (***).


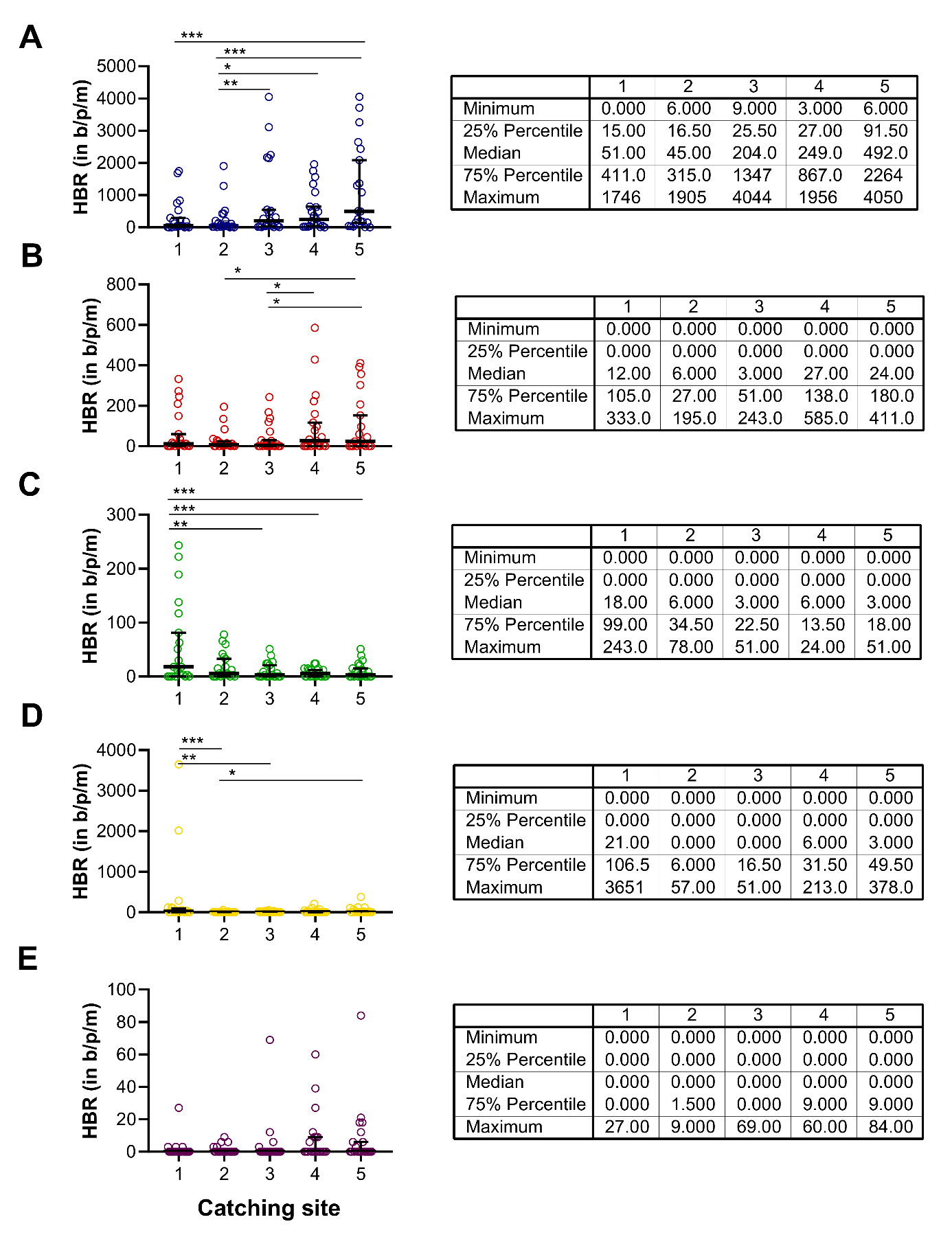


**Figure S3. Spatial heterogeneity in malaria vectors distribution in TOT.** A) Funestus Group, B) Maculatus Group, C) Leucosphyrus Group, D) Barbirostris Group, E) Annularis Group. Human-biting rate (HBR) estimates were collated per catching site for each entomological survey. The statistical significance of the differences observed between catching sites was assessed with the Friedman test and a post-hoc analysis. Dunn’s correction was applied for multiple comparisons. *p* <0.033 (*), <0.002 (**) and <0.001 (***).


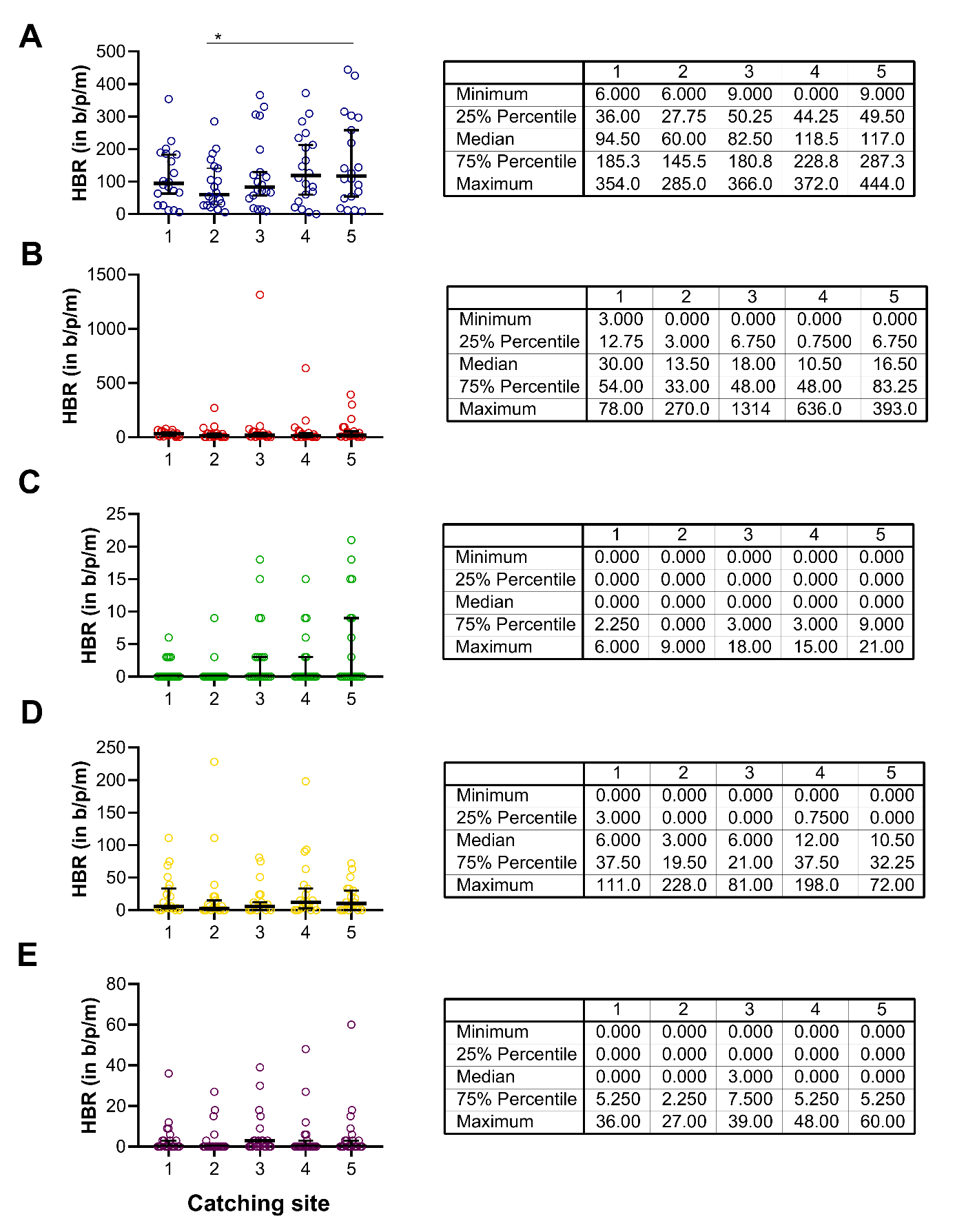


**Figure S4.** **Spatial heterogeneity in malaria vectors distribution in TPN.** A) Funestus Group, B) Maculatus Group, C) Leucosphyrus Group, D) Barbirostris Group, E) Annularis Group. Human-biting rate (HBR) estimates were collated per catching site for each entomological survey. The statistical significance of the differences observed between catching sites was assessed with the Friedman test and a post-hoc analysis. Dunn’s correction was applied for multiple comparisons. *p* <0.033 (*), <0.002 (**) and <0.001 (***).


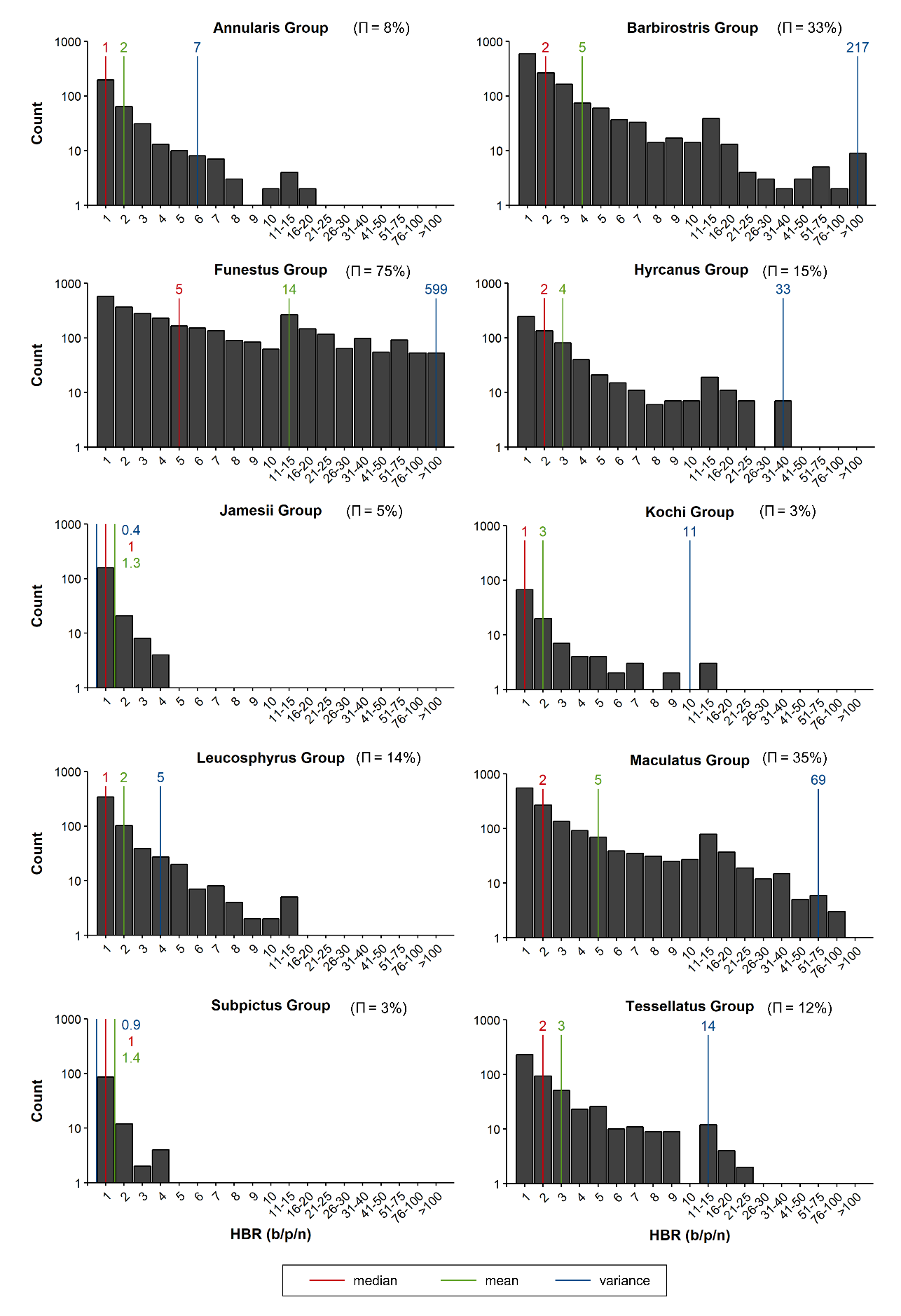


**Figure S5. Zero-truncated frequency distribution of the human-biting rate.** Individual estimates of the human-biting rate (HBR) are expressed as a number of bites/person/night. Mean, median and variance are indicated in green, red and blue respectively. The proportion of catches with positive HBR (Π) among the 4120 person-nights of collection is indicated into brackets.


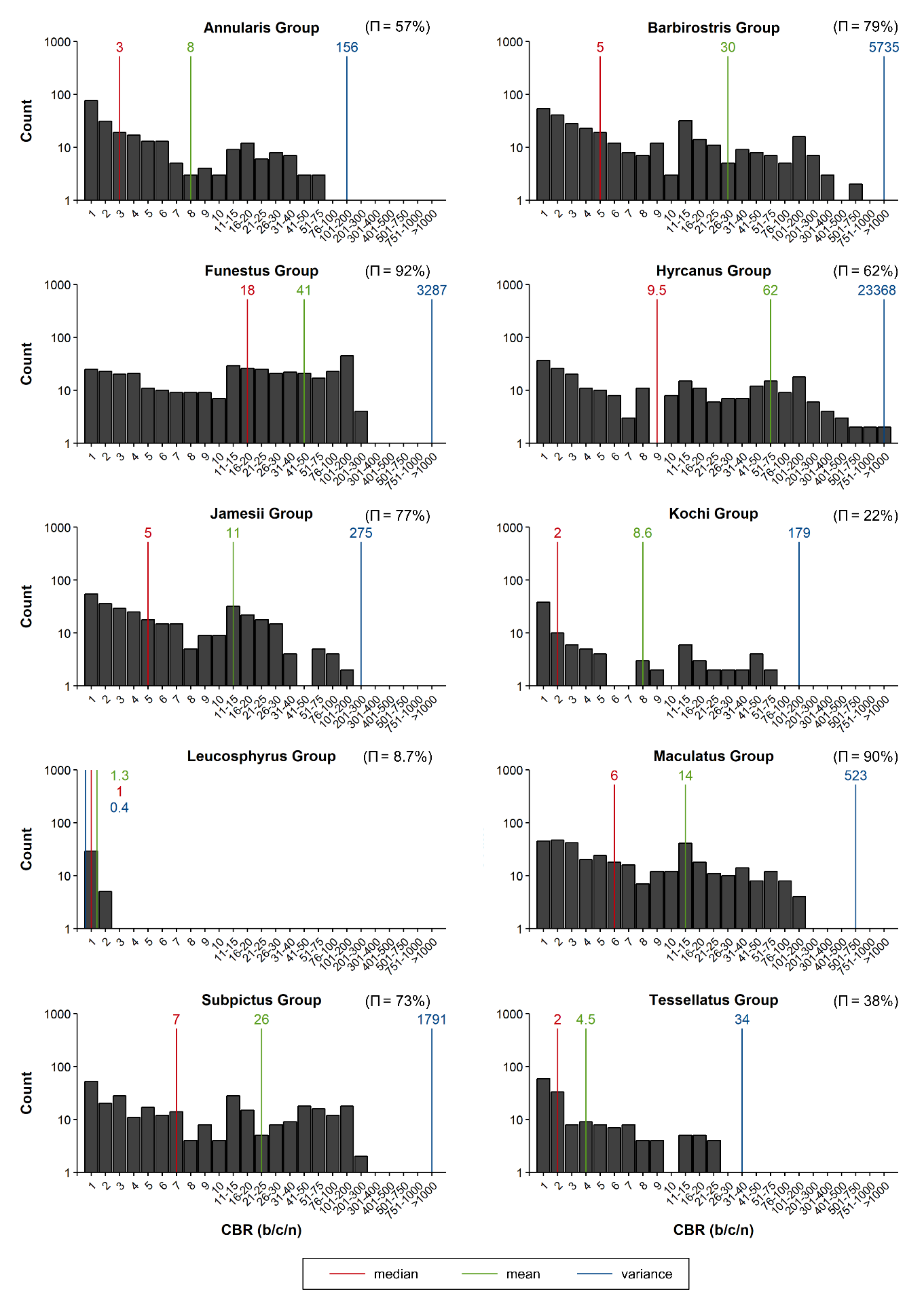


**Figure S6. Zero-truncated frequency distribution of the cow-biting rate.** Individual estimates of the cow-biting rate (CBR) are expressed as a number of bites/cow/night. Mean, median and variance are indicated in green, red and blue respectively. The proportion of catches with positive CBR (Π) among the 412 cow-nights of collection is indicated into brackets.


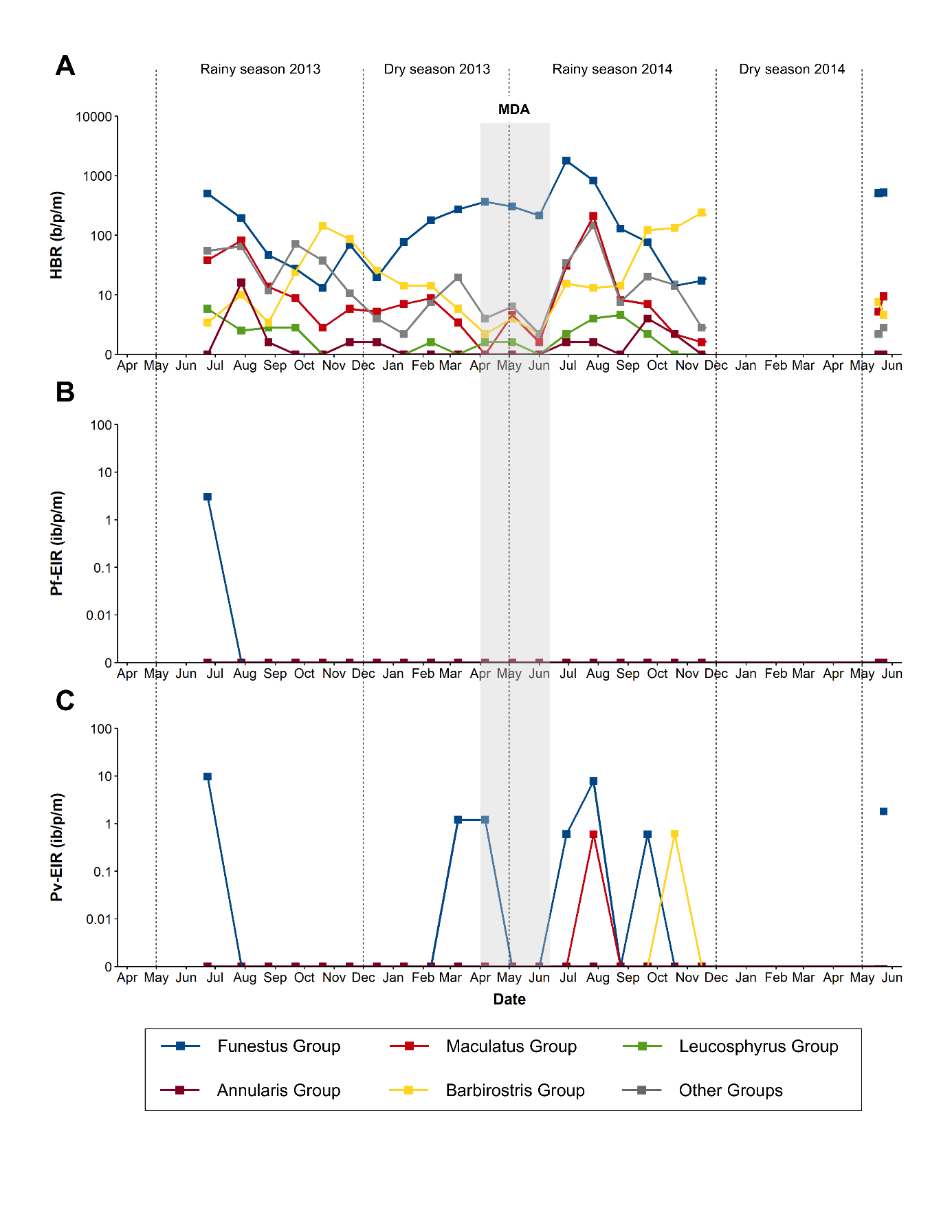
**Figure S7. Dynamics of the entomological indices in HKT**. A) Human-biting rate (HBR) expressed in number of bites /person /month; B) *Plasmodium falciparum* entomological inoculation rate (Pf-EIR) expressed in number of infective bites /person /month; C) *Plasmodium vivax* entomological inoculation rate (Pv-EIR) expressed in number of infective bites /person /month. Mass drug administration (MDA) is indicated by the grey panel.


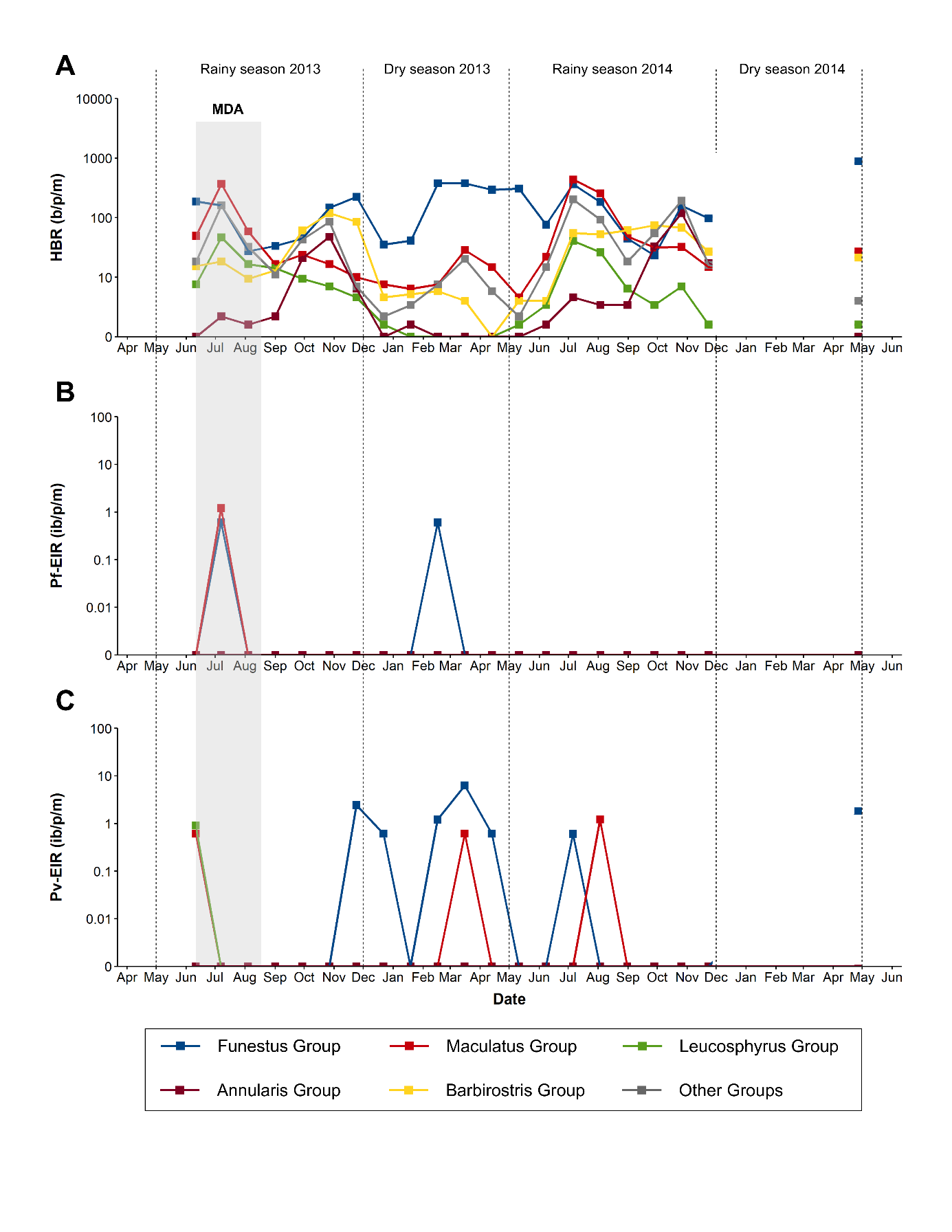
 **Figure S8. Dynamics of the entomological indices in KNH.** A) Human-biting rate (HBR) expressed in number of bites /person /month; B) *Plasmodium falciparum* entomological inoculation rate (Pf-EIR) expressed in number of infective bites /person /month; C) *Plasmodium vivax* entomological inoculation rate (Pv-EIR) expressed in number of infective bites /person /month. Mass drug administration (MDA) is indicated by the grey panel.


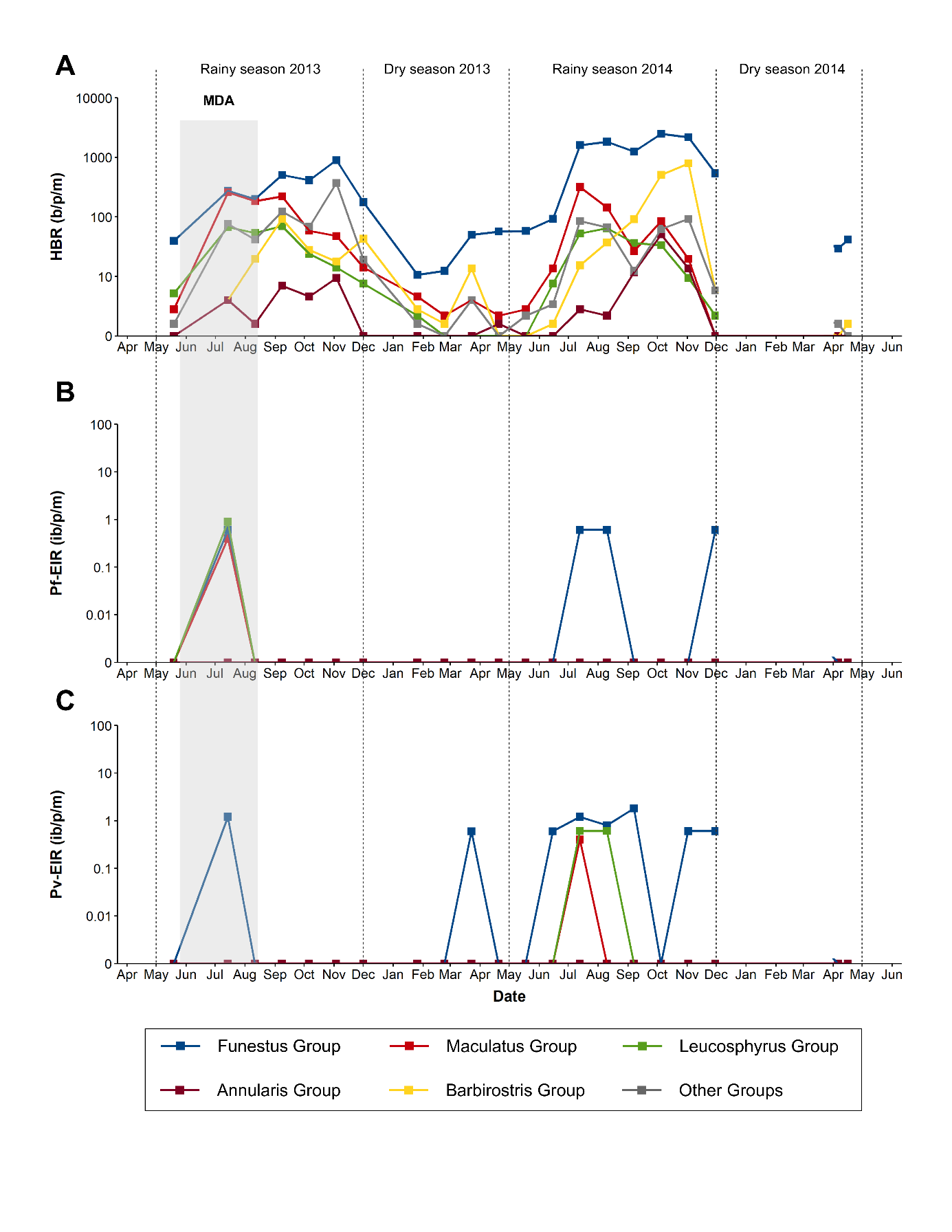
 **Figure S9. Dynamics of the entomological indices in TOT.** A) Human-biting rate (HBR) expressed in number of bites /person /month; B) *Plasmodium falciparum* entomological inoculation rate (Pf-EIR) expressed in number of infective bites /person /month; C) *Plasmodium vivax* entomological inoculation rate (Pv-EIR) expressed in number of infective bites /person /month. Mass drug administration (MDA) is indicated by the grey panel.


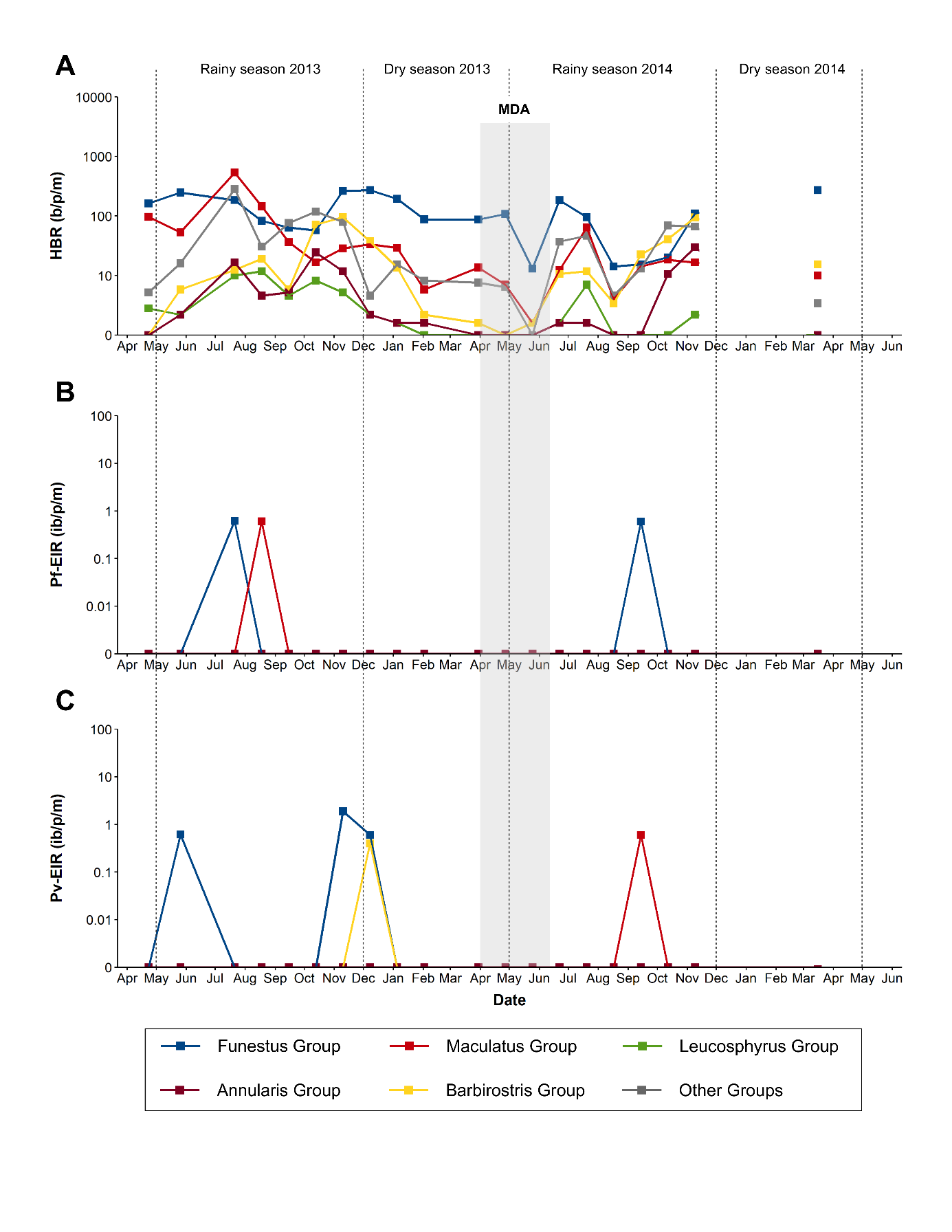
 **Figure S10. Dynamics of the entomological indices in TPN.** A) Human-biting rate (HBR) expressed in number of bites /person /month; B) *Plasmodium falciparum* entomological inoculation rate (Pf-EIR) expressed in number of infective bites /person /month; C) *Plasmodium vivax* entomological inoculation rate (Pv-EIR) expressed in number of infective bites /person /month. Mass drug administration (MDA) is indicated by the grey panel.


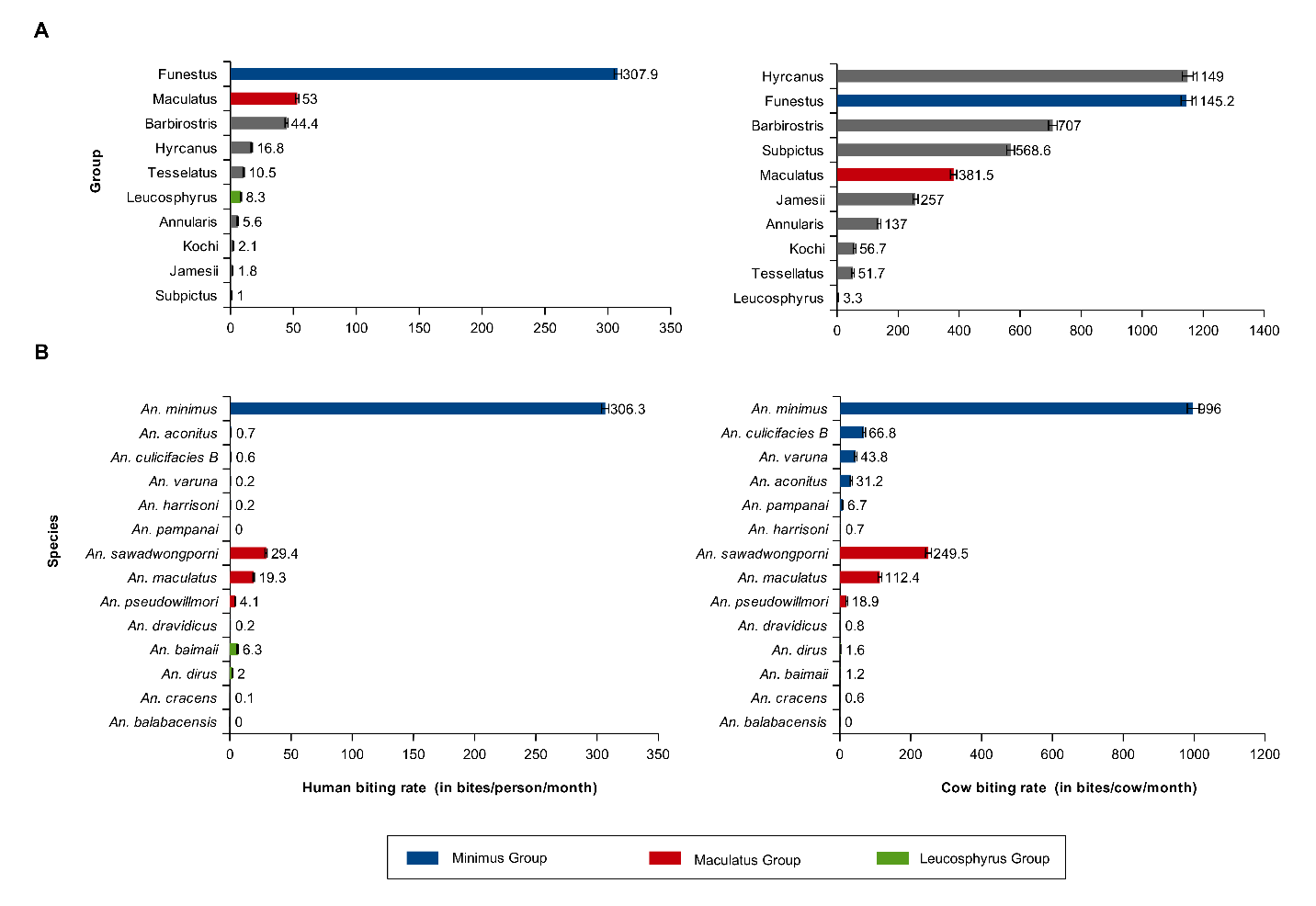
 **Figure S11. Human-biting rate (HBR) and cow-biting rate (CBR) of Anopheles mosquitoes.** A) HBR and CBR are presented at the Group level. B) HBR and CBR are presented at the species level.

**
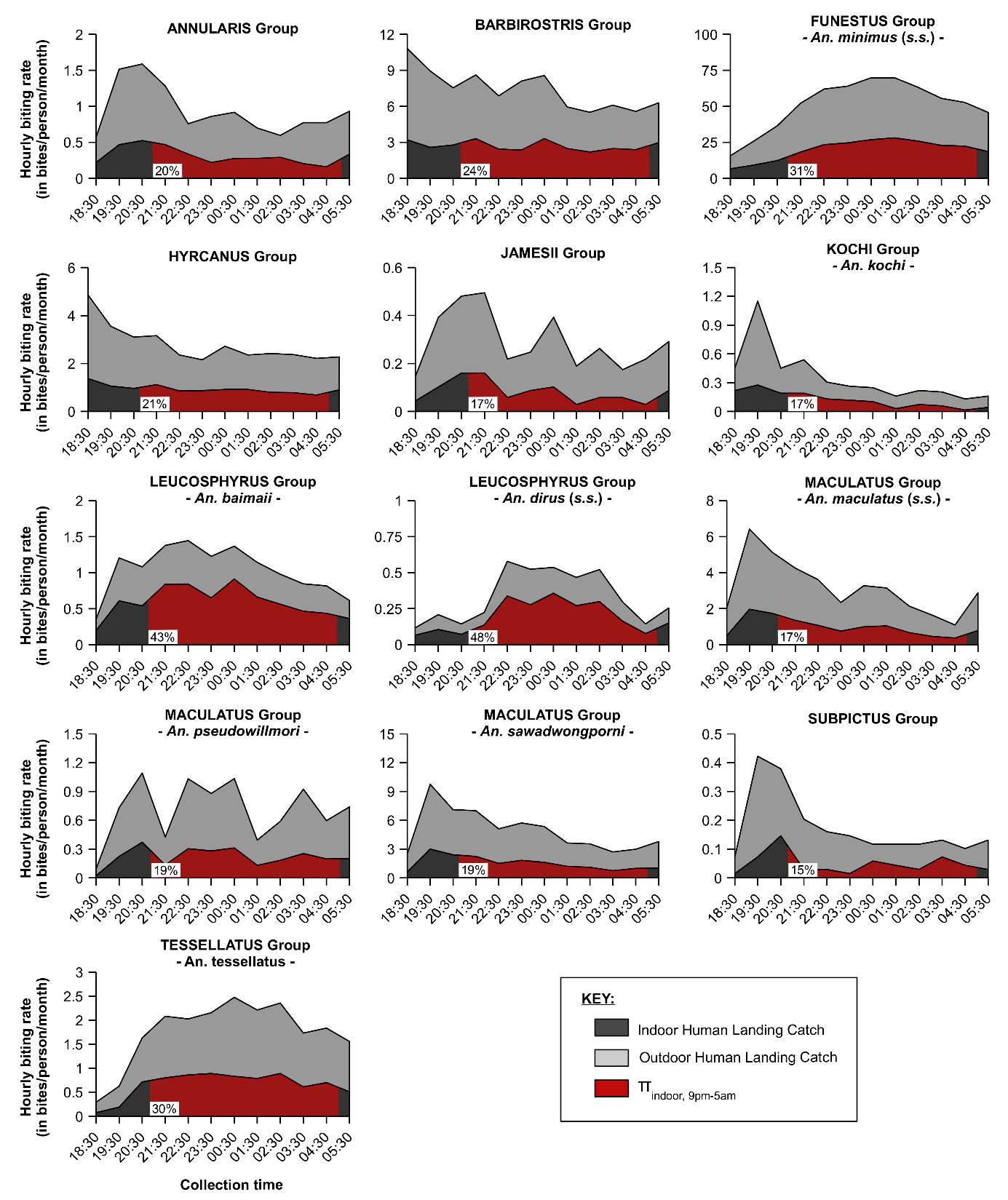
 Figure S12. Hourly biting pattern of *Anopheles* mosquitoes collected by human-landing catch.** Π_indoors, 9pm-5am_: proportion of the specimens collected indoors between 09:00 pm and 05:00 am.


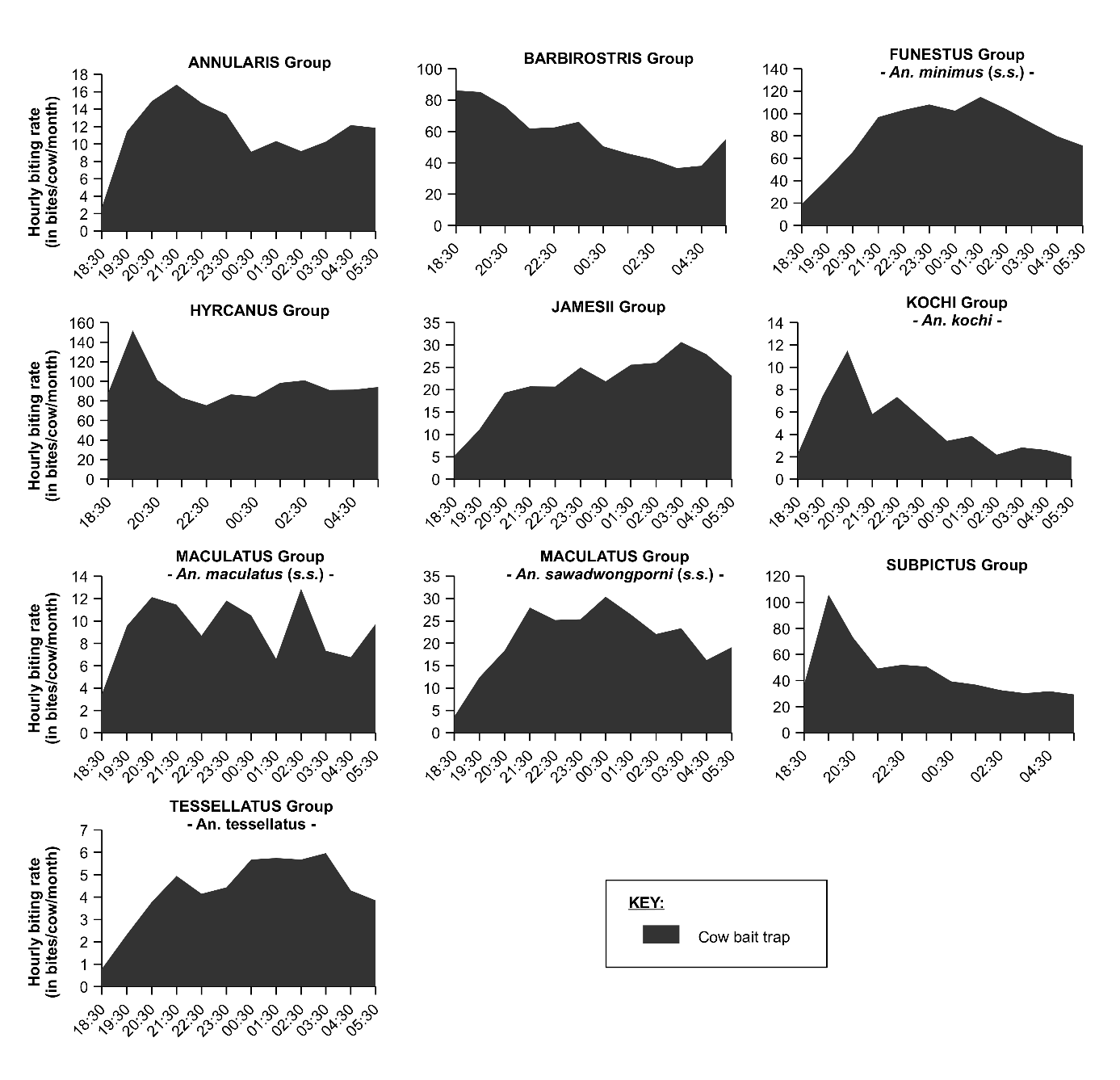
 **Figure S13. Hourly biting pattern of *Anopheles* mosquitoes collected by cow-bait trap.**

**Table S1. Planning of the entomological surveys.**

| **Village** | **Survey** | **Date** | **Person-nights** | **Cow-nights** |
| --- | --- | --- | --- | --- |
| HKT | 1 | 2013-06-23 | 50 | 5 |
| HKT | 2 | 2013-07-28 | 20 | 2 |
| HKT | 3 | 2013-08-25 | 50 | 5 |
| HKT | 4 | 2013-09-22 | 50 | 5 |
| HKT | 5 | 2013-10-20 | 50 | 5 |
| HKT | 6 | 2013-11-17 | 50 | 5 |
| HKT | 7 | 2013-12-15 | 50 | 5 |
| HKT | 8 | 2014-01-12 | 50 | 5 |
| HKT | 9 | 2014-02-09 | 50 | 5 |
| HKT | 10 | 2014-03-09 | 50 | 5 |
| HKT | 11 | 2014-04-06 | 50 | 5 |
| HKT | 12 | 2014-05-04 | 50 | 5 |
| HKT | 13 | 2014-06-01 | 50 | 5 |
| HKT | 14 | 2014-06-29 | 50 | 5 |
| HKT | 15 | 2014-07-27 | 50 | 5 |
| HKT | 16 | 2014-08-24 | 50 | 5 |
| HKT | 17 | 2014-09-21 | 50 | 5 |
| HKT | 18 | 2014-10-19 | 50 | 5 |
| HKT | 19 | 2014-11-16 | 50 | 5 |
| HKT | 20 | 2015-05-18 | 50 | 5 |
| HKT | 21 | 2015-05-23 | 50 | 5 |
| KNH | 1 | 2013-06-11 | 50 | 5 |
| KNH | 2 | 2013-07-07 | 50 | 5 |
| KNH | 3 | 2013-08-04 | 50 | 5 |
| KNH | 4 | 2013-09-01 | 50 | 5 |
| KNH | 5 | 2013-09-29 | 50 | 5 |
| KNH | 6 | 2013-10-27 | 50 | 5 |
| KNH | 7 | 2013-11-24 | 50 | 5 |
| KNH | 8 | 2013-12-22 | 50 | 5 |
| KNH | 9 | 2014-01-19 | 50 | 5 |
| KNH | 10 | 2014-02-16 | 50 | 5 |
| KNH | 11 | 2014-03-16 | 50 | 5 |
| KNH | 12 | 2014-04-13 | 50 | 5 |
| KNH | 13 | 2014-05-11 | 50 | 5 |
| KNH | 14 | 2014-06-08 | 50 | 5 |
| KNH | 15 | 2014-07-06 | 50 | 5 |
| KNH | 16 | 2014-08-03 | 50 | 5 |
| KNH | 17 | 2014-08-31 | 50 | 5 |
| KNH | 18 | 2014-09-28 | 50 | 5 |
| KNH | 19 | 2014-10-26 | 50 | 5 |
| KNH | 20 | 2014-11-23 | 50 | 5 |
| KNH | 21 | 2015-04-27 | 50 | 5 |
| TOT | 1 | 2013-05-19 | 50 | 5 |
| TOT | 2 | 2013-07-14 | 50 | 5 |
| TOT | 3 | 2013-08-11 | 50 | 5 |
| TOT | 4 | 2013-09-08 | 50 | 5 |
| TOT | 5 | 2013-10-06 | 50 | 5 |
| TOT | 6 | 2013-11-03 | 50 | 5 |
| TOT | 7 | 2013-12-01 | 50 | 5 |
| TOT | 8 | 2014-01-26 | 50 | 5 |
| TOT | 9 | 2014-02-23 | 50 | 5 |
| TOT | 10 | 2014-03-23 | 50 | 5 |
| TOT | 11 | 2014-04-20 | 50 | 5 |
| TOT | 12 | 2014-05-18 | 50 | 5 |
| TOT | 13 | 2014-06-15 | 50 | 5 |
| TOT | 14 | 2014-07-13 | 50 | 5 |
| TOT | 15 | 2014-08-10 | 50 | 5 |
| TOT | 16 | 2014-09-07 | 50 | 5 |
| TOT | 17 | 2014-10-05 | 50 | 5 |
| TOT | 18 | 2014-11-02 | 50 | 5 |
| TOT | 19 | 2014-11-30 | 50 | 5 |
| TOT | 20 | 2015-04-06 | 50 | 5 |
| TOT | 21 | 2015-04-16 | 50 | 5 |
| TPN | 1 | 2013-04-23 | 50 | 5 |
| TPN | 2 | 2013-05-26 | 50 | 5 |
| TPN | 3 | 2013-07-21 | 50 | 5 |
| TPN | 4 | 2013-08-18 | 50 | 5 |
| TPN | 5 | 2013-09-15 | 50 | 5 |
| TPN | 6 | 2013-10-13 | 50 | 5 |
| TPN | 7 | 2013-11-10 | 50 | 5 |
| TPN | 8 | 2013-12-08 | 50 | 5 |
| TPN | 9 | 2014-01-05 | 50 | 5 |
| TPN | 10 | 2014-02-02 | 50 | 5 |
| TPN | 11 | 2014-03-02 | 0 | 0 |
| TPN | 12 | 2014-03-30 | 50 | 5 |
| TPN | 13 | 2014-04-27 | 50 | 5 |
| TPN | 14 | 2014-05-25 | 50 | 5 |
| TPN | 15 | 2014-06-22 | 50 | 5 |
| TPN | 16 | 2014-07-20 | 50 | 5 |
| TPN | 17 | 2014-08-17 | 50 | 5 |
| TPN | 18 | 2014-09-14 | 50 | 5 |
| TPN | 19 | 2014-10-12 | 50 | 5 |
| TPN | 20 | 2014-11-09 | 50 | 5 |
| TPN | 21 | 2015-03-16 | 50 | 5 |

**Table S2. Results of the molecular identification of sibling species in the Funestus, Maculatus and Leucosphyrus Groups presented per village.**

| **Group** | **Species** | **HKT** | | | **KNH** | | | **TOT** | | | **TPN** | | |
| --- | --- | --- | --- | --- | --- | --- | --- | --- | --- | --- | --- | --- | --- |
|  |  | **n/N** | **p** | **95%CI** | **n/N** | **p** | **95%CI** | **n/N** | **p** | **95%CI** | **n/N** | **p** | **95%CI** |
| Funestus | *An. aconitus* | 11/1608 | 0.7 | 0.3-1.2 | 9/1048 | 0.9 | 0.4-1.6 | 2/1096 | 0.2 | 0-0.7 | 27/1089 | 2.5 | 1.6-3.6 |
|  | *An. culicifacies B* | 3/1608 | 0.2 | 0-0.5 | 6/1048 | 0.6 | 0.2-1.2 | 4/1096 | 0.4 | 0.1-0.9 | 83/1089 | 7.6 | 6.1-9.4 |
|  | *An. harrisoni* | 0/1608 | 0 | - | 1/1048 | 0.1 | 0-0.5 | 1/1096 | 0.1 | 0-0.5 | 1/1089 | 0.1 | 0-0.5 |
|  | *An. minimus* (*s.s.*) | 1548/1608 | 96.3 | 95.2-97.1 | 1026/1048 | 97.9 | 96.8-98.7 | 1085/1096 | 99 | 98.2-99.5 | 960/1089 | 88.2 | 86.1-90 |
|  | *An. pampanai* | 2/1608 | 0.1 | 0-0.4 | 0/1048 | 0 | - | 2/1096 | 0.2 | 0-0.7 | 5/1089 | 0.5 | 0.1-1.1 |
|  | *An. varuna* | 43/1608 | 2.7 | 1.9-3.6 | 5/1048 | 0.5 | 0.2-1.1 | 1/1096 | 0.1 | 0-0.5 | 12/1089 | 1.1 | 0.6-1.9 |
| Maculatus | *An. dravidicus* | 3/568 | 0.5 | 0.1-1.5 | 2/814 | 0.2 | 0-0.9 | 0/1096 | 0 | - | 4/1002 | 0.4 | 0.1-1 |
|  | *An. maculatus* (*s.s.*) | 116/568 | 20.4 | 17.2-24 | 404/814 | 49.6 | 46.1-53.1 | 87/587 | 14.8 | 12-18 | 369/1002 | 36.8 | 33.8-39.9 |
|  | *An. pseudowillmori* | 37/568 | 6.5 | 4.6-8.9 | 73/814 | 9 | 7.1-11.1 | 21/587 | 3.6 | 2.2-5.4 | 57/1002 | 5.7 | 4.3-7.3 |
|  | *An. sawadwongporni* | 411/568 | 72.4 | 68.5-76 | 334/814 | 41 | 37.6-44.5 | 478/587 | 81.4 | 78-84.5 | 571/1002 | 57 | 53.9-60.1 |
| Leucosphyrus | *An. baimaii* | 25/28 | 89.3 | 71.8-97.7 | 249/257 | 96.9 | 94-98.6 | 323/542 | 59.6 | 55.3-63.8 | 56/62 | 90.3 | 80.1-96.4 |
|  | *An. balabacensis* | 0/28 | 0 | - | 0/257 | 0 | - | 2/542 | 0.4 | 0-1.3 | 1/62 | 1.6 | 0-8.7 |
|  | *An. cracens* | 2/28 | 7.1 | 0.9-23.5 | 2/257 | 0.8 | 0.1-2.8 | 5/542 | 0.9 | 0.3-2.1 | 1/62 | 1.6 | 0-8.7 |
|  | *An. dirus* (*s.s.*) | 0/28 | 0 | - | 5/257 | 1.9 | 0.6-4.5 | 211/542 | 38.9 | 34.8-43.2 | 3/62 | 4.8 | 1-13.5 |

**Table S3. Results of the molecular identification of sibling species in the Funestus, Maculatus and Leucosphyrus Groups presented per season.**

| **Group** | **Species** | **Dry** | | | **Rainy** | | |
| --- | --- | --- | --- | --- | --- | --- | --- |
|  |  | **n/N** | **p** | **95%CI** | **n/N** | **p** | **95%CI** |
| Funestus | *An. aconitus* | 22/1649 | 1.3 | 0.8-2 | 27/3190 | 0.8 | 0.6-1.2 |
|  | *An. culicifacies B* | 83/1649 | 5 | 4-6.2 | 13/3190 | 0.4 | 0.2-0.7 |
|  | *An. harrisoni* | 1/1649 | 0.1 | 0-0.3 | 2/3190 | 0.1 | 0-0.2 |
|  | *An. minimus* (*s.s.*) | 1521/1649 | 92.2 | 90.8-93.5 | 3098/3190 | 97.1 | 96.5-97.7 |
|  | *An. pampanai* | 5/1649 | 0.3 | 0.1-0.7 | 4/3190 | 0.1 | 0-0.3 |
|  | *An. varuna* | 16/1649 | 1 | 0.6-1.6 | 45/3190 | 1.4 | 1-1.9 |
| Maculatus | *An. dravidicus* | 1/1205 | 0.1 | 0-0.5 | 8/1764 | 0.5 | 0.2-0.9 |
|  | *An. maculatus* (*s.s.*) | 466/1205 | 38.7 | 35.9-41.5 | 510/1764 | 28.9 | 26.8-31.1 |
|  | *An. pseudowillmori* | 39/1205 | 3.2 | 2.3-4.4 | 149/1764 | 8.4 | 7.2-9.8 |
|  | *An. sawadwongporni* | 698/1205 | 57.9 | 55.1-60.7 | 1096/1764 | 62.1 | 59.8-64.4 |
| Leucosphyrus | *An. baimaii* | 4/14 | 28.6 | 8.4-58.1 | 649/873 | 74.3 | 71.3-77.2 |
|  | *An. balabacensis* | 0/14 | 0 | - | 3/873 | 0.3 | 0.1-1 |
|  | *An. cracens* | 4/14 | 28.6 | 8.4-58.1 | 6/873 | 0.7 | 0.3-1.5 |
|  | *An. dirus* (*s.s.*) | 5/14 | 35.7 | 12.8-64.9 | 214/873 | 24.5 | 21.7-27.5 |

**Table S4. List of *Plasmodium* positive samples.**

| **ID** | **Date** | **Village** | **Collection Method** | **Group** | ***Anopheles spp.*** | ***Plasmodium spp.*** |
| --- | --- | --- | --- | --- | --- | --- |
| 1 | 2013-05-30 | TPN | HLC | Funestus | *An. minimus* (*s.s.*) | PV |
| 2 | 2013-06-12 | KNH | HLC | Maculatus | *An. sawadwongporni* | PV |
| 3 | 2013-06-13 | KNH | HLC | Leucosphyrus | *An. baimaii* | PV |
| 4 | 2013-06-23 | HKT | HLC | Funestus | *An. minimus* (*s.s.*) | PV |
| 5 | 2013-06-23 | HKT | HLC | Funestus | *An. minimus* (*s.s.*) | PF/PV |
| 6 | 2013-06-23 | HKT | HLC | Funestus | *An. minimus* (*s.s.*) | PV |
| 7 | 2013-06-23 | HKT | HLC | Funestus | *An. minimus* (*s.s.*) | PV |
| 8 | 2013-06-24 | HKT | HLC | Funestus | *An. minimus* (*s.s.*) | PV |
| 9 | 2013-06-24 | HKT | HLC | Funestus | *An. minimus* (*s.s.*) | PV |
| 10 | 2013-06-24 | HKT | HLC | Funestus | *An. minimus* (*s.s.*) | PV |
| 11 | 2013-06-25 | HKT | HLC | Funestus | *An. minimus* (*s.s.*) | PV |
| 12 | 2013-06-25 | HKT | HLC | Funestus | *An. minimus* (*s.s.*) | PV |
| 13 | 2013-06-25 | HKT | HLC | Funestus | *An. minimus* (*s.s.*) | PV |
| 14 | 2013-06-25 | HKT | HLC | Funestus | *An. minimus* (*s.s.*) | PV |
| 15 | 2013-06-26 | HKT | HLC | Funestus | *An. minimus* (*s.s.*) | PV |
| 16 | 2013-06-26 | HKT | HLC | Funestus | *An. minimus* (*s.s.*) | PV |
| 17 | 2013-06-26 | HKT | HLC | Funestus | *An. minimus* (*s.s.*) | PF |
| 18 | 2013-06-26 | HKT | HLC | Funestus | *An. minimus* (*s.s.*) | PF |
| 19 | 2013-06-26 | HKT | HLC | Funestus | *An. minimus* (*s.s.*) | PV |
| 20 | 2013-06-27 | HKT | HLC | Funestus | *An. minimus* (*s.s.*) | PV |
| 21 | 2013-06-27 | HKT | HLC | Funestus | *An. minimus* (*s.s.*) | PF |
| 22 | 2013-06-27 | HKT | HLC | Funestus | *An. minimus* (*s.s.*) | PF |
| 23 | 2013-06-27 | HKT | HLC | Funestus | *An. minimus* (*s.s.*) | PV |
| 24 | 2013-07-09 | KNH | HLC | Maculatus | *An. sawadwongporni* | PF |
| 25 | 2013-07-10 | KNH | HLC | Maculatus | *An. sawadwongporni* | PF |
| 26 | 2013-07-11 | KNH | HLC | Funestus | *An. minimus* (*s.s.*) | PF |
| 27 | 2013-07-15 | TOT | HLC | Maculatus | *An. sawadwongporni* | PF |
| 28 | 2013-07-15 | TOT | HLC | Leucosphyrus | *An. dirus* (*s.s.*) | PF |
| 29 | 2013-07-16 | TOT | HLC | Funestus | *An. minimus* (*s.s.*) | PV |
| 30 | 2013-07-16 | TOT | HLC | Funestus | *An. minimus* (*s.s.*) | PF |
| 31 | 2013-07-17 | TOT | HLC | Funestus | *An. minimus* (*s.s.*) | PV |
| 32 | 2013-07-22 | TPN | HLC | Funestus | *An. minimus* (*s.s.*) | PF |
| 33 | 2013-08-19 | TPN | HLC | Maculatus | *An. maculatus* (*s.s.*) | PF |
| 34 | 2013-10-16 | TPN | HLC | Maculatus | not determined | PJ |
| 35 | 2013-11-12 | TPN | HLC | Funestus | *An. minimus* (*s.s.*) | PV |
| 36 | 2013-11-12 | TPN | HLC | Funestus | *An. minimus* (*s.s.*) | PV |
| 37 | 2013-11-13 | TPN | HLC | Funestus | *An. minimus* (*s.s.*) | PV |
| 38 | 2013-11-13 | TPN | HLC | Funestus | *An. minimus* (*s.s.*) | PJ |
| 39 | 2013-11-24 | KNH | HLC | Funestus | *An. minimus* (*s.s.*) | PV |
| 40 | 2013-11-24 | KNH | CBT | Funestus | *An. minimus* (*s.s.*) | PV |
| 41 | 2013-11-28 | KNH | HLC | Funestus | *An. minimus* (*s.s.*) | PV |
| 42 | 2013-11-28 | KNH | HLC | Funestus | *An. minimus* (*s.s.*) | PV |
| 43 | 2013-11-28 | KNH | HLC | Funestus | *An. minimus* (*s.s.*) | PV |
| 44 | 2013-12-10 | TPN | HLC | Funestus | *An. aconitus* | PV |
| 45 | 2013-12-11 | TPN | HLC | Barbirostris | not determined | PV |
| 46 | 2013-12-25 | KNH | HLC | Funestus | *An. minimus* (*s.s.*) | PV |
| 47 | 2014-02-18 | KNH | HLC | Funestus | *An. minimus* (*s.s.*) | PV |
| 48 | 2014-02-18 | KNH | HLC | Funestus | *An. minimus* (*s.s.*) | PF |
| 49 | 2014-02-20 | KNH | HLC | Funestus | *An. minimus* (*s.s.*) | PV |
| 50 | 2014-03-11 | HKT | HLC | Funestus | *An. minimus* (*s.s.*) | PV |
| 51 | 2014-03-13 | HKT | HLC | Funestus | *An. minimus* (*s.s.*) | PV |
| 52 | 2014-03-17 | KNH | HLC | Funestus | *An. minimus* (*s.s.*) | PV |
| 53 | 2014-03-18 | KNH | HLC | Funestus | *An. minimus* (*s.s.*) | PV |
| 54 | 2014-03-18 | KNH | HLC | Funestus | not determined | PV |
| 55 | 2014-03-18 | KNH | HLC | Funestus | *An. minimus* (*s.s.*) | PV |
| 56 | 2014-03-18 | KNH | HLC | Funestus | *An. minimus* (*s.s.*) | PV |
| 57 | 2014-03-18 | KNH | HLC | Funestus | *An. minimus* (*s.s.*) | PV |
| 58 | 2014-03-20 | KNH | HLC | Funestus | *An. minimus* (*s.s.*) | PV |
| 59 | 2014-03-20 | KNH | HLC | Maculatus | *An. sawadwongporni* | PV |
| 60 | 2014-03-20 | KNH | HLC | Funestus | *An. minimus* (*s.s.*) | PV |
| 61 | 2014-03-20 | KNH | HLC | Funestus | *An. minimus* (*s.s.*) | PV |
| 62 | 2014-03-20 | KNH | HLC | Funestus | *An. minimus* (*s.s.*) | PV |
| 63 | 2014-03-23 | TOT | HLC | Funestus | *An. minimus* (*s.s.*) | PV |
| 64 | 2014-04-07 | HKT | HLC | Funestus | *An. minimus* (*s.s.*) | PV |
| 65 | 2014-04-07 | HKT | HLC | Funestus | not determined | PV |
| 66 | 2014-04-15 | KNH | HLC | Funestus | *An. minimus* (*s.s.*) | PV |
| 67 | 2014-06-17 | TOT | HLC | Funestus | *An. minimus* (*s.s.*) | PV |
| 68 | 2014-06-30 | HKT | HLC | Funestus | *An. minimus* (*s.s.*) | PV |
| 69 | 2014-07-07 | KNH | HLC | Funestus | *An. minimus* (*s.s.*) | PV |
| 70 | 2014-07-09 | KNH | HLC | Leucosphyrus | *An. baimaii* | PJ |
| 71 | 2014-07-13 | TOT | HLC | Maculatus | *An. sawadwongporni* | PV |
| 72 | 2014-07-16 | TOT | HLC | Funestus | *An. minimus* (*s.s.*) | PF |
| 73 | 2014-07-16 | TOT | HLC | Funestus | *An. minimus* (*s.s.*) | PV |
| 74 | 2014-07-17 | TOT | HLC | Leucosphyrus | *An. baimaii* | PV |
| 75 | 2014-07-17 | TOT | HLC | Funestus | *An. minimus* (*s.s.*) | PV |
| 76 | 2014-07-27 | HKT | HLC | Funestus | *An. minimus* (*s.s.*) | PV |
| 77 | 2014-07-27 | HKT | HLC | Funestus | *An. minimus* (*s.s.*) | PV |
| 78 | 2014-07-28 | HKT | HLC | Funestus | *An. minimus* (*s.s.*) | PV |
| 79 | 2014-07-29 | HKT | HLC | Funestus | *An. minimus* (*s.s.*) | PV |
| 80 | 2014-07-29 | HKT | HLC | Funestus | *An. minimus* (*s.s.*) | PV |
| 81 | 2014-07-29 | HKT | HLC | Funestus | *An. minimus* (*s.s.*) | PV |
| 82 | 2014-07-29 | HKT | HLC | Funestus | *An. minimus* (*s.s.*) | PV |
| 83 | 2014-07-29 | HKT | HLC | Funestus | not determined | PV |
| 84 | 2014-07-30 | HKT | HLC | Funestus | *An. minimus* (*s.s.*) | PV |
| 85 | 2014-07-31 | HKT | HLC | Funestus | *An. minimus* (*s.s.*) | PV |
| 86 | 2014-07-31 | HKT | HLC | Funestus | *An. minimus* (*s.s.*) | PV |
| 87 | 2014-07-31 | HKT | HLC | Maculatus | *An. sawadwongporni* | PV |
| 88 | 2014-07-31 | HKT | HLC | Funestus | *An. minimus* (*s.s.*) | PV |
| 89 | 2014-07-31 | HKT | HLC | Funestus | *An. minimus* (*s.s.*) | PV |
| 90 | 2014-08-03 | KNH | HLC | Maculatus | not determined | PV |
| 91 | 2014-08-07 | KNH | HLC | Maculatus | *An. maculatus* (*s.s.*) | PV |
| 92 | 2014-08-11 | TOT | HLC | Funestus | not determined | PJ |
| 93 | 2014-08-12 | TOT | HLC | Funestus | *An. minimus* (*s.s.*) | PV |
| 94 | 2014-08-14 | TOT | HLC | Leucosphyrus | *An. dirus* (*s.s.*) | PV |
| 95 | 2014-08-14 | TOT | HLC | Funestus | *An. minimus* (*s.s.*) | PF |
| 96 | 2014-09-08 | TOT | HLC | Funestus | *An. minimus* (*s.s.*) | PV |
| 97 | 2014-09-09 | TOT | HLC | Funestus | *An. minimus* (*s.s.*) | PV |
| 98 | 2014-09-10 | TOT | HLC | Funestus | *An. minimus* (*s.s.*) | PV |
| 99 | 2014-09-14 | TPN | HLC | Maculatus | *An. maculatus* (*s.s.*) | PV |
| 100 | 2014-09-16 | TPN | HLC | Funestus | *An. minimus* (*s.s.*) | PF |
| 101 | 2014-09-25 | HKT | HLC | Funestus | *An. minimus* (*s.s.*) | PV |
| 102 | 2014-10-19 | HKT | CBT | Funestus | *An. minimus* (*s.s.*) | PV |
| 103 | 2014-10-20 | HKT | HLC | Barbirostris | not determined | PV |
| 104 | 2014-11-03 | TOT | HLC | Funestus | *An. minimus* (*s.s.*) | PV |
| 105 | 2014-11-30 | TOT | HLC | Funestus | *An. minimus* (*s.s.*) | PV |
| 106 | 2014-12-04 | TOT | HLC | Funestus | *An. minimus* (*s.s.*) | PF |
| 107 | 2015-04-28 | KNH | HLC | Funestus | *An. minimus* (*s.s.*) | PV |
| 108 | 2015-05-01 | KNH | HLC | Funestus | *An. minimus* (*s.s.*) | PV |
| 109 | 2015-05-01 | KNH | HLC | Funestus | *An. minimus* (*s.s.*) | PV |
| 110 | 2015-05-18 | HKT | HLC | Funestus | *An. minimus* (*s.s.*) | PJ |
| 111 | 2015-05-25 | HKT | HLC | Funestus | *An. minimus* (*s.s.*) | PV |
| 112 | 2015-05-27 | HKT | HLC | Funestus | *An. minimus* (*s.s.*) | PV |
| 113 | 2015-05-27 | HKT | HLC | Funestus | *An. minimus* (*s.s.*) | PV |

**CBT:** cow-bait trap**, HLC:** human-landing catch, **PF**: *Plasmodium falciparum,* **PJ**: *P. juxtanucleare*, **PV**: *P. vivax*.

**Table S5. Descriptive statistics of the sporozoite load in naturally infected malaria vectors from hotspot villages located in the Thailand-Myanmar border area.**

| **Plasmodium species** | **Parameter** | **Value of the parameter for the indicated *Anopheles* Group** | | | | |
| --- | --- | --- | --- | --- | --- | --- |
|  |  | **Minimus** | **Maculatus** | **Dirus** | **Barbirostris** | **Total** |
| Pf | N | 11 | 4 | 1 | 0 | 16 |
|  | Geom. mean | 74 | 13 | 6 | - | 41 |
|  | 95%CI | 18-222 | 7-23 | - | - | 14-98 |
|  | Minimum | 10 | 6 | - | - | 6 |
|  | 1st quartile | 16 | 8.25 | - | - | 10.75 |
|  | Median | 26 | 13.5 | - | - | 21 |
|  | 3rd quartile | 328 | 21 | - | - | 89.25 |
|  | Maximum | 9234 | 30 | - | - | 9234 |
| Pv | N | 78 | 7 | 3 | 2 | 90 |
|  | Geom. mean | 186 | 26 | 897 | 26 | 162 |
|  | 95%CI | 101-318 | 14-54 | 118-22352 | 20-34 | 94-267 |
|  | Minimum | 6 | 4 | 36 | 20 | 4 |
|  | 1st quartile | 36 | 20 | 1485 | 23.5 | 28.5 |
|  | Median | 57 | 26 | 2934 | 27 | 51.5 |
|  | 3rd quartile | 472.2 | 50 | 4884 | 30.5 | 398 |
|  | Maximum | 517500 | 92 | 6834 | 34 | 517500 |
| Total | N | 89 | 11 | 4 | 2 | 106 |
|  | Geom. mean | 166 | 20 | 257 | 26 | 131 |
|  | 95%CI | 100-270 | 12-36 | 15-4478 | 20-34 | 79-206 |
|  | Minimum | 6 | 4 | 6 | 20 | 4 |
|  | 1st quartile | 27 | 13.5 | 28.5 | 23.5 | 25.25 |
|  | Median | 55 | 20 | 1485 | 27 | 45 |
|  | 3rd quartile | 482 | 33 | 3909 | 30.5 | 258.2 |
|  | Maximum | 517500 | 92 | 6834 | 34 | 517500 |

**NA**: not applicable; **N**: number of specimens; **95%CI**: 95% confidence interval; **Geom. mean**: geometric mean.
