# Supplementary material for: Entomological determinants of malaria transmission in Kayin state, Eastern Myanmar: A 24-month longitudinal study in four villages
Source: Wellcome Open Res. 2019 Jun 17;3:109. Originally published 2018 Aug 31. [Version 4] doi: 10.12688/wellcomeopenres.14761.4 (PMC6544137; doi:10.12688/wellcomeopenres.14761.4)
Supplement: Supplementary file 2 [file wellcomeopenres-3-16720-s0001.tgz › c50e5229-5dc5-49af-951f-f6efc284ff8d_Supplementary_File_2_revised.docx]

**DNA extraction:**

Samples were crushed in 200μL of cetyltrimethylammonium bromide (CTAB) solution 2% (TrisHCl pH = 8, 20mM; EDTA 10mM; NaCL, 1.4 mM; N-cetyl-N,N,N-trimethyl ammonium bromide 2%) with a TissueLyser II ™ (Qiagen) set on 29mvt/sec for 3 minutes. Samples were then warmed at 65°C for 5 minutes and 200μl of chloroform were added. The organic phase was collected and DNA was precipitated with 200μL of isopropanol. After centrifugation at 20,000 g for 15 minutes, the pellet was washed twice with 200µL of 70% ethanol and suspended in 40 μL of PCR grade water.

**AS-PCR assay for the Funestus Group**

Closely related species from to the Funestus Group were discriminated using an AS-PCR assay adapted from Garros *et al.^[[1]](#footnote-1)^*. The PCR mix was composed of 1X Goldstar™ DNA polymerase (Eurogentec, Seraing, Belgium) and 400 nM of each primer (**Table 1**). The PCR was conducted in a total reaction volume of 25 μl (4 μl of DNA template and 21 μl of PCR mix). The thermocycling protocol consisted in an initial activation step of 1 min at 94 °C, followed by 40 amplification cycles of 20 s at 94 °C, 20 s at 45 °C and 30 s at 72 °C. The length of the PCR product was determined by gel electrophoresis in 2% agarose for 70 min at 120V.

Table 1. Sequence of the primers used for the molecular identification of closely related species from the Funestus Group (adapted from Garros *et al*. 2004).

| **Primer name** | **Sequence** | **Product length (in bp)** | **Species** |
| --- | --- | --- | --- |
| ITS2A **†** | 5’-TGTGAACTGCAGGACACAT-3’ | - | - |
| MIA **‡** | 5’-CCCGTGCGACTTGACGA-3’ | 310 | *An. minimus* (*s.s*.) |
| MIC **‡** | 5’-GTTCATTCAGCAACATCAGT-3’ | 180 | *An. harrisoni* |
| ACO **‡** | 5’-ACAGCGTGTACGTCCAGT-3’ | 200 | *An. aconitus* |
| PAM **‡** | 5’-TGTACATCGGCCGGGGTA-3’ | 90 | *An. pampanai* |
| VAR **‡** | 5’-TTGACCACTTTCGACGCA-3’ | 260 | *An. varuna* |

**†** Universal forward primer

**‡** Species specific reverse primers

**AS-PCR assay for the Maculatus Group**

Closely related species from to the Maculatus Group were discriminated using an AS-PCR assay adapted from Walton *et al.^[[2]](#footnote-2)^*. The PCR mix was composed of 1X Goldstar™ DNA polymerase (Eurogentec, Seraing, Belgium) and 400 nM of each primer (**Table 2**). The PCR was conducted in a total reaction volume of 25 μl (4 μl of DNA template and 21 μl of PCR mix). The thermocycling protocol consisted in an initial activation step of 1 min at 94 °C, followed by 40 amplification cycles of 20 s at 94 °C, 20 s at 55 °C and 30 s at 72 °C. The length of the PCR product was determined by gel electrophoresis in 2% agarose for 70 min at 120V.

Table 2. Sequence of the primers used for the molecular identification of closely related species from the Maculatus Group (adapted from Walton *et al*. 2007).

| **Primer name** | **Sequence** | **Product length (in bp)** | **Species** |
| --- | --- | --- | --- |
| 5.8F**†** | 5’-TGTGAACTGCAGGACACAT-3’ | - | - |
| MAC**‡** | 5’-CCCGTGCGACTTGACGA-3’ | 310 | *An. maculatus* (*s.s*.) |
| PSEU**‡** | 5’-GTTCATTCAGCAACATCAGT-3’ | 180 | *An. pseudowillmori* |
| SAW**‡** | 5’-ACAGCGTGTACGTCCAGT-3’ | 200 | *An. sawadwongporni* |
| K**‡** | 5’-TGTACATCGGCCGGGGTA-3’ | 90 | *An. rampae* |
| DRAV**‡** | 5’-TTGACCACTTTCGACGCA-3’ | 260 | *An. dravidicus* |

**†** Universal forward primer

**‡** Species specific reverse primers

**AS-PCR assay for the Leucosphyrus Group**

Closely related species from to the Leucosphyrus Group were discriminated using an AS-PCR assay adapted from Walton *et al.^[[3]](#footnote-3)^*. The PCR mix was composed of 1X Goldstar™ DNA polymerase (Eurogentec, Seraing, Belgium) and 400 nM of each primer (**Table 3**). The PCR was conducted in a total reaction volume of 25 μl (4 μl of DNA template and 21 μl of PCR mix). The thermocycling protocol consisted in an initial activation step of 1 min at 94 °C, followed by 40 amplification cycles of 20 s at 94 °C, 20 s at 55 °C and 30 s at 72 °C. The length of the PCR product was determined by gel electrophoresis in 2% agarose for 70 min at 120V.

Table 3. Sequence of the primers used for the molecular identification of closely related species from the Leucosphyrus Group (adapted from Walton *et al*. 2007).

| **Primer name** | **Sequence** | **Product length (in bp)** | **Species** |
| --- | --- | --- | --- |
| D-AC**†** | 5’-CACAGCGACTCCACACG-3’ | - | - |
| D-B**‡** | 5’-CGGGATATGGGTCGGCC-3’ | 562/353 | *An. dirus* (*s.s.*) / *An. scanloni* |
| D-D**‡** | 5’-GCGCGGGACCGTCCGTT-3’ | 514 | *An. cracens* |
| D-F**‡** | 5’-AACGGCGGTCCCCTTTG-3’ | 306 | *An. baimaii* |
| D-AC**‡** | 5’-CACAGCGACTCCACACG-3’ | 223 | *An. nemophilous* |

**†** Universal forward primer

**‡** Species specific reverse primers

**Molecular identification by sequencing of the ITS2 DNA marker**

Amplification of ITS2 was performed using the primer pair ITS2A (5'-TGT GAA CTG CAG GAC ACA T-3') and ITS2B (5'-ATG CTT AAA TTY AGG GGG T-3') described by Beebe *et al.^[[4]](#footnote-4)^*. The PCR mix was composed of 1X Goldstar™ DNA polymerase (Eurogentec, Seraing, Belgium) and 400 nM of each primer. The PCR was conducted in a total reaction volume of 25 μl (4 μl of DNA template and 21 μl of PCR mix). The thermocycling protocol consisted in an initial activation step of 1 min at 94 °C, followed by 40 amplifcation cycles of 20 s at 94 °C, 20 s at 51 °C and 30 s at 72 °C. The PCR product was sequenced by Macrogen™ (Seoul, South-Korea) using the ITS2A primer. The sequence was analysed using the blastn algorithm of the online BLAST™ software^[[5]](#footnote-5)^ in order to determine the corresponding species.

***Plasmodium* detection using quantitative real-time PCR**

DNA extracts were screened for the presence of *Plasmodium* using a quantitative real-time PCR (qrtPCR) assay that targets the 18S ssuRNA genes with the primer pair PL1473F18/PL1679R18 described by Mangold *et al.^[[6]](#footnote-6)^* (**Table 4**). The presence of *Plasmodium* sporozoites was confirmed in all positive samples by a second qrt-PCR assay that amplifies the COX I and COX III genes of *P. falciparum* and *P. vivax* respectively, using the primer pairs Pf1/Pf2 and Pv1/Pv2 described by Cunha *et al.^[[7]](#footnote-7)^* (**Table 4**).

Table 4. Sequence of the primers used for *Plasmodium* detection (adapted from Mangold *et al*., 2007 and Cunha *et al*, 2009).

| **Primer name** | **Sequence** |
| --- | --- |
| PL1473F18 | 5’-TAA CGA ACG AGA TCT TAA-3’ |
| PL1679R18 | 5’-GTT CCT CTA AGA AGC TTT-3’ |
| Pf1 | 5’-CCT GCA TTA ACA TCA TTA TAT GGT ACA TCT-3’ |
| Pf2 | 5’-GAT TAA CAT TCT TGA TGA AGT AAT GAT AAT ACC TT-3’ |
| Pv1 | 5’-AAG TGT TGT ATG GGC TCA TCA TAT G-3’ |
| Pv2 | 5’-CAA AAT GGA AAT GAG CGA TTA CAT-3’ |

The PCR mix was composed of 1X HOT FIREPol™ EvaGreen™ qPCR Mix Plus (Solis BioDyne, Tartu, Estonia) and 250 nM (screening assay) or 300 nM (confirmation assay) of each primer. The PCR was conducted in a total reaction volume of 10 μl (1 μL of DNA template and 9 μL of PCR mix) on a CFX-96 Touch™ real-time detection system (Biorad). The thermocycling protocol consisted in an initial activation step of 1 min at 94°C, followed by 45 amplification cycles of 15 sec at 95°C, 20 sec at 54°C (screening assay) or 20 sec at 60°C (confirmation assay), and 20 sec at 72°C. The melt curve of the PCR product was determined as follow: 95°C for 15 sec, 68°C for 1 minute, 80°C for 15 sec, 60°C for 15 s sec, then 60°C to 90°C with an Increment of 0.2°C /sec.

For both screening and confirmation assays, calibrated suspensions of *P. falciparum* and *P. vivax* sporozoites were used in order to verify the efficacy of the PCR reaction, the limit of detection (LOD) and to set-up standard curves for the absolute quantitation of sporozoite load in infected specimens^[[8]](#footnote-8)^. The efficacy of the screening assay was 97% and 101% on *P. falciparum* and *P. vivax* respectively. The efficacy of the confirmation assay was 84% and 92% on *P. falciparum* and *P. vivax* respectively. The LOD of the screening assay was estimated at 60 and 36 sporozoites / mosquito for *P. falciparum* and *P. vivax* respectively. The LOD of the confirmation assay was estimated at 6 and 3.6 sporozoites / mosquito for *P. falciparum* and *P. vivax* respectively.

In case confirmation assay yielded negative results, the PCR product was sequenced by Macrogen™ (Seoul, South-Korea) using the PL1473F18 primer. The sequence was analysed using the blastn algorithm of the online BLAST™ software ^[[9]](#footnote-9)^ in order to determine the specificity of the signal.

1. Garros, C., L. L. Koekemoer, M. Coetzee, M. Coosemans, and S. Manguin. "A Single Multiplex Assay to Identify Major Malaria Vectors within the African Anopheles Funestus and the Oriental An. Minimus Groups." [In eng]. Am J Trop Med Hyg 70, no. 6 (Jun 2004): 583-90. [↑](#footnote-ref-1)
2. Walton, C., P. Somboon, S. M. O'Loughlin, S. Zhang, R. E. Harbach, Y. M. Linton, B. Chen, et al. "Genetic Diversity and Molecular Identification of Mosquito Species in the Anopheles Maculatus Group Using the Its2 Region of Rdna." [In eng]. Infect Genet Evol 7, no. 1 (Jan 2007): 93-102. [↑](#footnote-ref-2)
3. Walton, C., J. M. Handley, C. Kuvangkadilok, F. H. Collins, R. E. Harbach, V. Baimai, and R. K. Butlin. "Identification of Five Species of the Anopheles Dirus Complex from Thailand, Using Allele-Specific Polymerase Chain Reaction." [In eng]. Med Vet Entomol 13, no. 1 (Feb 1999): 24-32. [↑](#footnote-ref-3)
4. Beebe, N. W., and A. Saul. "Discrimination of All Members of the Anopheles Punctulatus Complex by Polymerase Chain Reaction--Restriction Fragment Length Polymorphism Analysis." [In eng]. Am J Trop Med Hyg 53, no. 5 (Nov 1995): 478-81. [↑](#footnote-ref-4)
5. McWilliam, H., W. Li, M. Uludag, S. Squizzato, Y. M. Park, N. Buso, A. P. Cowley, and R. Lopez. "Analysis Tool Web Services from the Embl-Ebi." [In eng]. Nucleic Acids Res 41, no. 2 (Jul 2013): W597-600. [↑](#footnote-ref-5)
6. Mangold, K. A., R. U. Manson, E. S. Koay, L. Stephens, M. Regner, R. B. Thomson, Jr., L. R. Peterson, and K. L. Kaul. "Real-Time Pcr for Detection and Identification of Plasmodium Spp." [In eng]. J Clin Microbiol 43, no. 5 (May 2005): 2435-40. [↑](#footnote-ref-6)
7. Cunha, M. G., T. S. Medina, S. G. Oliveira, A. N. Marinho, M. M. Povoa, and A. K. Ribeiro-dos-Santos. "Development of a Polymerase Chain Reaction (Pcr) Method Based on Amplification of Mitochondrial Dna to Detect Plasmodium Falciparum and Plasmodium Vivax." [In eng]. Acta Trop 111, no. 1 (2009): 35-8. [↑](#footnote-ref-7)
8. Chaumeau, V., C. Andolina, B. Fustec, N. Tuikue Ndam, C. Brengues, S. Herder, D. Cerqueira, et al. "Comparison of the Performances of Five Primer Sets for the Detection and Quantification of Plasmodium in Anopheline Vectors by Real-Time Pcr." [In eng]. PLoS One 11, no. 7 (2016): e0159160. [↑](#footnote-ref-8)
9. McWilliam, H., W. Li, M. Uludag, S. Squizzato, Y. M. Park, N. Buso, A. P. Cowley, and R. Lopez. "Analysis Tool Web Services from the Embl-Ebi." [In eng]. Nucleic Acids Res 41, no. 2 (Jul 2013): W597-600. [↑](#footnote-ref-9)
