# Supplementary material for: Entomological determinants of malaria transmission in Kayin state, Eastern Myanmar: A 24-month longitudinal study in four villages
Source: Wellcome Open Res. 2019 Jun 17;3:109. Originally published 2018 Aug 31. [Version 4] doi: 10.12688/wellcomeopenres.14761.4 (PMC6544137; doi:10.12688/wellcomeopenres.14761.4)
Supplement: Peer review history

# Peer review: Reviewer response for version 3

**DOI:** 10.21956/wellcomeopenres.16652.r35477

**Published:** 2019-05-29

**Authors:** Catherine Bourgouin

**Affiliations:** 1 Functional Genetics of Infectious Diseases, Institut Pasteur, CNRS UMR 2000, Paris, France

I approve the current version after fixing three minor points:

- Page 8: "The proportion of malaria vectors collected indoors between 09:00 pm and 05:00 am was 29% (range= 15–48% according to the species). Conversely, 64% of the infected specimens were collected out of doors, before 09:00 pm and/or after 05:00 am (Figure 5)".

**Based on the data in Figure 5, 29% should be 36%; the last sentence should be rephrased as for instance: "**Conversely, 64% of the infected specimens were collected **either** out of doors, **or **before 09:00 pm and after 05:00 am.

- Page 13: "In this study, only 36% of the Plasmodium-infected mosquitoes were collected indoors between 09:00 pm and 05:00 am (when and where people are expected to sleep under a bed-net). This proportion might have been **underestimated** as malaria vectors were already active at 06:00 pm and/or at 06:00 am, suggesting that the exposure stretched out of the collection time".

**Underestimated, should actually be "overestimated". **

- Page 14, end of the 2nd paragraph: "In this regard, targeted sequencing of **16S ribosomal RNA genes** detected in DNA extracts from blood-fed specimens is a promising tool for the determination of blood-meal sources in wild populations of malaria vectors"

**Change "16S ribosomal RNA genes" to "mammalian mt16S ribosomal RNA genes", as "16S ribosomal RNA genes" alone can be confusing as related to bacterial genomes.**

I have read this submission. I believe that I have an appropriate level of expertise to confirm that it is of an acceptable scientific standard.

# Response

**Published:** 2019-06-02

**Authors:** Victor Chaumeau

**Affiliations:** Shoklo Malaria Research Unit, Thailand

We thank to the reviewer for approving the version 3 of our manuscript.

- Page 8: "The proportion of malaria vectors collected indoors between 09:00 pm and 05:00 am was 29% (range= 15–48% according to the species). Conversely, 64% of the infected specimens were collected out of doors, before 09:00 pm and/or after 05:00 am (Figure 5)". Based on the data in Figure 5, 29% should be 36%; the last sentence should be rephrased as for instance: "Conversely, 64% of the infected specimens were collected either out of doors, or before 09:00 pm and after 05:00 am.

Page 8, 29% refers to the proportion of malaria vectors collected indoors between 09:00 pm and 05:00 am independently of their infection status, whereas 64% refers only to the proportion of *Plasmodium*-infected specimen collected outdoors, or indoors early in the evening or early in the morning. Therefore, we do not wish to change the number cited in the main text. The sentence has been reworded in the revised manuscript as per reviewer suggestion.

- Page 13: "In this study, only 36% of the Plasmodium-infected mosquitoes were collected indoors between 09:00 pm and 05:00 am (when and where people are expected to sleep under a bed-net). This proportion might have been underestimated as malaria vectors were already active at 06:00 pm and/or at 06:00 am, suggesting that the exposure stretched out of the collection time". Underestimated, should actually be "overestimated".

In the revised manuscript, typo page 13 “underestimated” was changed to "overestimated" as per reviewer’s suggestion.

- Page 14, end of the 2nd paragraph: "In this regard, targeted sequencing of 16S ribosomal RNA genes detected in DNA extracts from blood-fed specimens is a promising tool for the determination of blood-meal sources in wild populations of malaria vectors". Change "16S ribosomal RNA genes" to "mammalian mt16S ribosomal RNA genes", as "16S ribosomal RNA genes" alone can be confusing as related to bacterial genomes.

Page 14 “16S ribosomal RNA genes” was changed to “mammalian mt16S ribosomal RNA genes” as per reviewer’s suggestion.

# Peer review: Reviewer response for version 2

**DOI:** 10.21956/wellcomeopenres.16466.r34777

**Published:** 2019-03-20

**Authors:** Catherine Bourgouin

**Affiliations:** 1 Functional Genetics of Infectious Diseases, Institut Pasteur, CNRS UMR 2000, Paris, France

The authors took into consideration a large number of points that were addressed by the two reviewers. Several very informative data as tables or graphs have been included, principally as sup data. This to my opinion reinforces the quality and amount of work behind this investigation.

 Nevertheless, I still have some concern with 2 parts:

- Page 10, paragraph starting with “average values”. I do not follow the reasoning from “The lowest HBR measured on a single collector.." until the end of this paragraph.

What exactly do you mean by “mean HBR measured on single collectors”?

 Usually when using HCL, more than one collector is involved per class (indoor versus outdoor) located in different houses to avoid bias linked to the person or the surrounding of the house. We therefore combined data from several collectors within a village over a define time scale, as here month.

 It may be just a matter of presenting, arguing the data presented in this paragraph. I feel it may need some clarification.

- The discussion section:

I feel this could be largely improved by including conclusion of the different mosquito species biting humans and which one appears the most relevant local vector, if any, based on *Plasmodium* carriage. This is clearly presented in the sup figures (S7-S10).

 Similarly, the huge work presented in sup data S11-S12, could argue toward “shift intervention control” and “residual malaria transmission”. Except in the second § on page 14, was a mosquito species ever mentioned.

 On page 13, the 1 ^st^ § discussed more the longevity of the mosquitoes for being unable to transmit Pf, than the abundance component. Is the shorter life cycle of *P. vivax* a dogma that could explain its persistence in mosquitoes, rather than the presence of the best competent mosquito species the year around? And relapse in people? Interesting old data, but more recent that the Boyd book, on *P.vivax *and mosquitoes could be found as for instance “Am. J. Trop. Med. Hyg., 37(2), 1987, pp. 241 -245" ^1^. In addition, duration **of** *Plasmodium* sporogonic development is highly dependent on the T° whether the parasite species is *falciparum* or *vivax.*

 Additionally, discussing whether the MDA had some impact or not on parasite prevalence in mosquitoes could be interesting, if the sample sizes are enough informative.

 Overall, I found some parts of the discussion too long and to some extent not linked enough to the data, ie the § on residual malaria.

 Additional important comments:

- Not noticed in my former review, the ITS2 (laboratory procedure & sup data) is not a mitochondrial marker, but a rDNA marker.
- Tables 3 to 6: The “N” does not correspond to the same class between HBR, SI and EIR, please add proper legend.
- Page 8, last §: just after or in the title, it would be wise to add the analysis is done at the village scale. You could also make a brief comment on whether there is or not significant differences for the Pf/Pf EIR between villages.

Additional minor comments:

- When using “falciparum malaria”; it is a standard to not use italics “ *falciparum*”, same for vivax malaria
- Page 3 : The last 2 sentences of the last “intro” § are somehow redundant.
- Page 3 “study villages”: could you give the range for how high is the submicroscopic malaria prevalence?
- Figure 1 legend: consider using plural for “village, house and site”
- M&M:
  - qrtPCR: for molecular biologist it should be only qPCR, as qrtPCR might be confusing with Reverse Transcription and quantitative PCR (RT-qPCR)
  - to avoid going to the publication you cited, it would be simple to add “Mangold, targeting the *Plasmodium* 18S” and Cunha, *Plasmodium* mitochondrial targets, as indeed mentioned in the sup file 2.
- In the tables and in some additional places in the text: should not that be conventional to write numbers as 42,283 instead of 42283, as an example?
- In different places, I think that, as an example,: “malaria vector **S** ecology” should be “malaria vector ecology”.
- Few typos: page 3 last 2 sentences, 2 ^nd^ §: indo **orr**esidual &th **ee**cology, Page 4 th **eS**hoklo, “internal transcrib **ED** spacer”.
- Page 12 first §: “20% of the collectors received approximatively 50% of the bites and of the infective bites" => "20% of the collectors received approximatively 50% of the bites AS WELL AS 50% of the infective bites". Correct?
- Sup file 2: description of the PCR procedures for species identification: As the basic PCR is similar in terms of primer concentration, DNA template and final volume, I suggest to simplify and used a single table for the primers used adding a column with the specific temperature for primer annealing.

I have read this submission. I believe that I have an appropriate level of expertise to confirm that it is of an acceptable scientific standard, however I have significant reservations, as outlined above.

## References

[1] KasapHKasapMDemirhanOAlptekinD: Development of Plasmodium vivax in Anopheles superpictus under experimental conditions. *Am J Trop Med Hyg*.1987;37(2) :241-5 331068110.4269/ajtmh.1987.37.241

# Response

**Published:** 2019-04-26

**Authors:** Victor Chaumeau

**Affiliations:** Shoklo Malaria Research Unit, Thailand

We thank to the reviewer for her additional comments on the manuscript. Response to point specific comments are given below:

- **Page 10, paragraph starting with “average values”. I do not follow the reasoning from “The lowest HBR measured on a single collector.." until the end of this paragraph. What exactly do you mean by “mean HBR measured on single collectors” ? Usually when using HCL, more than one collector is involved per class (indoor versus outdoor) located in different houses to avoid bias linked to the person or the surrounding of the house. We therefore combined data from several collectors within a village over a define time scale, as here month. It may be just a matter of presenting, arguing the data presented in this paragraph. I feel it may need some clarification.**

The value of the entomological indices are longitudinal and spatially hierarchized. Therefore it is possible to describe the data at different level of aggregation ranging from hourly measurements on single individuals (mosquito collectors) to average values obtained by pooling the entire dataset. Mean values collated at the village and survey level are indeed useful to describe the data independently of bias linked to the person, the surrounding of the house or the night of collection, were reported in the first lines of the paragraph. Further insights into the characteristics of the entomological indices were be obtained by looking at different level of aggregation. For example, the range of HBR collated at the village and survey level was 13 to 2611 bites/person/month whereas the range of non-collated HBR estimates (number of mosquito collected during one night by a single mosquito collector) was 0 to 289 bites/person/night (8670 bites/person/month), further documenting the high abundance of malaria mosquitoes sometime observed in the area of the study. By looking at data collated at the collector level for the entire period of the study, we also observed that some mosquito collectors never captured *Plasmodium*-infected mosquitoes whereas 20% of the collectors received 50% of the infective bites, which would not have been shown by analyzing only aggregated data.

- **The discussion section: I feel this could be largely improved by including conclusion of the different mosquito species biting humans and which one appears the most relevant local vector, if any, based on Plasmodium carriage. This is clearly presented in the sup figures (S7-S10).**

We believe that the dynamic of malaria mosquitoes and their capacity to transmit malaria is extremely complex in the area of the study and that designating one species as the main malaria vector would be misleading. One characteristics of the study area is the high diversity of malaria vectors, which pullulate in different environments and at different time of the year. Although most of the *Plasmodium*-infected malaria mosquitoes were identified as *An. minimus s.s. *in the present study, we do not think that this species is *de facto *the main local malaria vectors. For example, An. dirus is an important vector in and around the forest and its contribution may have been underestimated in the present study.

- **Similarly, the huge work presented in sup data S11-S12, could argue toward “shift intervention control” and “residual malaria transmission”. **

The work presented in supplemental data Figures S10-11 is discussed in the paragraph “Residual malaria transmission” and “shift in vector control intervention”. We do not wish to add additional details in the discussion of the revised manuscript.

- **Except in the second § on page 14, was a mosquito species ever mentioned.**

Names of anopheles species were added at the very beginning of the discussion in the paragraph on the dynamics of entomological indices.

- **On page 13, the 1st § discussed more the longevity of the mosquitoes for being unable to transmit Pf, than the abundance component. Is the shorter life cycle of P. vivax a dogma that could explain its persistence in mosquitoes, rather than the presence of the best competent mosquito species the year around? And relapse in people? Interesting old data, but more recent that the Boyd book, on P.vivax and mosquitoes could be found as for instance “Am. J. Trop. Med. Hyg., 37(2), 1987, pp. 241 -245"1. In addition, duration of Plasmodium sporogonic development is highly dependent on the T° whether the parasite species is falciparum or vivax.**

The reviewer is right to mention that not only the longevity of malaria vectors can explain seasonal trends in malaria transmission. A sentence has been added at the end of the paragraph and the suggested bibliography was cited in the main text.

- **Additionally, discussing whether the MDA had some impact or not on parasite prevalence in mosquitoes could be interesting, if the sample sizes are enough informative.**

The impact of MDA on the entomological indices has been published separately in Chaumeau *et al.*, 2019 in the JID and we do not wish to add more details in the current manuscript.

- **Overall, I found some parts of the discussion too long and to some extent not linked enough to the data, ie the § on residual malaria.**

We believe that the paragraph on residual malaria transmission is important given the objective of the study. Direct link to the data are made in the paragraph on residual malaria transmission (eg. night biting pattern and corresponding distribution of infective bites indoors and outdoors over the course of the night, zoophagy, opportunistic blood-source selection). We wish to keep the paragraph on residual malaria transmission unchanged.

- **Not noticed in my former review, the ITS2 (laboratory procedure & sup data) is not a mitochondrial marker, but a rDNA marker.**

This mistake was corrected in the revised version of the manuscript.

- **Tables 3 to 6: The “N” does not correspond to the same class between HBR, SI and EIR, please add proper legend.**

Proper legend has been added in the Tables presenting entomological indices.

- **Page 8, last §: just after or in the title, it would be wise to add the analysis is done at the village scale. **

The data on the entomological indices are described at different level of aggregation (eg. average values on the entire data set, mean collated by village and by survey, non-collated estimates measured on individual mosquito collectors). We do not wish to change the title of this paragraph.

- **You could also make a brief comment on whether there is or not significant differences for the Pf/Pf EIR between villages.**

A detailed analysis of Pf- and Pv-EIRs in the context of malaria elimination has been published separately in Chaumeau *et al.* JID 2019. We do not wish to add more details in the current manuscript.

- **When using “falciparum malaria”; it is a standard to not use italics “falciparum”, same for vivax malaria**

“ *P. falciparum *malaria” p.13 was changed to falciparum malaria as per reviewer’s suggestion.

- **Page 3 : The last 2 sentences of the last “intro” § are somehow redundant.**

One sentence was removed as per reviewer’s suggestion.

- **Page 3 “study villages”: could you give the range for how high is the submicroscopic malaria prevalence?**

The range of submicroscopic malaria prevalence at the beginning of the study was cited in the main text. Additional information can be found in Landier *et al.* WOR 2017.

- **Figure 1 legend: consider using plural for “village, house and site”**

Figure 1 legend has been modified as per reviewer’s suggestion.

- **qrtPCR: for molecular biologist it should be only qPCR, as qrtPCR might be confusing with Reverse Transcription and quantitative PCR (RT-qPCR)**

qrtPCR was changed to qPCR in revised version of the manuscript.

- **to avoid going to the publication you cited, it would be simple to add “Mangold, targeting the Plasmodium 18S” and Cunha, Plasmodium mitochondrial targets, as indeed mentioned in the sup file 2.**

Names of PCR DNA targets were added in the main text as per reviewer’s suggestion.

- **In the tables and in some additional places in the text: should not that be conventional to write numbers as 42,283 instead of 42283, as an example?**

We do not wish to change number format in the revised manuscript.

- **In different places, I think that, as an example,: “malaria vectorS ecology” should be “malaria vector ecology”.**

Typos were corrected in the revised manuscript.

- **Few typos: page 3 last 2 sentences, 2nd §: indoorresidual &theecology, Page 4 theShoklo, “internal transcribED spacer”.**

Typos were corrected in the revised manuscript.

- **Page 12 first §: “20% of the collectors received approximatively 50% of the bites and of the infective bites" => "20% of the collectors received approximatively 50% of the bites AS WELL AS 50% of the infective bites". Correct?**

The sentence was modified to improve its readability by the reader.

- **Sup file 2: description of the PCR procedures for species identification: As the basic PCR is similar in terms of primer concentration, DNA template and final volume, I suggest to simplify and used a single table for the primers used adding a column with the specific temperature for primer annealing.**

We wish to keep the current outline of the supplementary file S2.

# Peer review: Reviewer response for version 2

**DOI:** 10.21956/wellcomeopenres.16466.r34776

**Published:** 2019-02-15

**Authors:** Lisa J. Reimer

**Affiliations:** 1 Department of Vector Biology, Liverpool School of Tropical Medicine, Liverpool, UK

No further comments.

I have read this submission. I believe that I have an appropriate level of expertise to confirm that it is of an acceptable scientific standard.

# Peer review: Reviewer response for version 1

**DOI:** 10.21956/wellcomeopenres.16087.r34267

**Published:** 2018-12-10

**Authors:** Lisa J. Reimer

**Affiliations:** 1 Department of Vector Biology, Liverpool School of Tropical Medicine, Liverpool, UK

This is a very comprehensive study which was completed to a high standard. The knowledge generated is essential to achieve malaria elimination in the sub-region. My two essential recommendations for revision include 1) a deeper description on vector ecology in this region and a summary of which control and prevention activities are currently in place. This background can draw from the work that was described previously in reference 17. The end of the introduction states “Numerous aspects of malaria vectors ecology and biology have not been documented and the characteristics of the entomological indices are not known precisely” but the reader would appreciate a full description of the current gaps in our knowledge. 2) There is a significant amount of data that has been collected and analysed, but this could be organised in a way that is much more accessible. For example a table that summarizes key attributes per vector group (if it is not always available by species) such as mean and range monthly biting rates, perhaps median biting times, EI, ZI, infection prevalence.

 Further specific comments are below:

1. This is described as a pilot study on targeted malaria elimination, however this is such a comprehensive entomological survey that it diminishes the significance of the work to call it a pilot.
2. Typo under entomological surveys: “mosquito sampling the using human landing catch”
3. It would be more intuitive to presented sporozoite prevalence rather than out of 1000 mosquitoes
4. Figure 4b and 5b – how is the HBR, EI and ZI calculated at the species level when a smaller proportion of samples were ID’d molecularly? Is it a proportion of those that were morphologically identified?
5. Figure 5 – what are the dashed lines for? It might be more useful to use the line to show a community mean index
6. Typo under “residual malaria transmission”: “35% of infection mosquitoes collected indoors between 9pm and 5pm”
7. Rephrase the final sentence “in order to address specific problematic in the context of malaria elimination”

I have read this submission. I believe that I have an appropriate level of expertise to confirm that it is of an acceptable scientific standard, however I have significant reservations, as outlined above.

# Response

**Published:** 2019-01-27

**Authors:** Victor Chaumeau

**Affiliations:** Shoklo Malaria Research Unit, Thailand

We are thankful to the reviewer for her useful feed-back on the manuscript. Part of the introduction was rewritten in order to provide the reader with more background information on malaria vectors, transmission settings and on-going elimination efforts in the area of the study. A full description of the current gaps in our knowledge on vector ecology and biology would be difficult to present in the format of this research article. We instead referred the reader to the review of Sinka *et al. *that summarize key gaps for each dominant malaria vectors in the Asia-Pacific region. We tried to improve the presentation of the results by keeping only the key tables and figures in the main text and by adding more supplementary figures and tables in the supplementary data. Unfortunately, in was not possible to summarize in one table the statistics of all key indices, together with the raw numbers and confidence intervals. Answer to specific comments are below:

- This is described as a pilot study on targeted malaria elimination, however this is such a comprehensive entomological survey that it diminishes the significance of the work to call it a pilot.

The wording pilot has been removed from the revised manuscript.

- Typo under entomological surveys: “mosquito sampling the using human landing catch”

The typo has been corrected.

- It would be more intuitive to presented sporozoite prevalence rather than out of 1000 mosquitoe.

Sporozoite prevalence was expressed as percentages rather than out of 1000 mosquitoes.

- Figure 4b and 5b – how is the HBR, EI and ZI calculated at the species level when a smaller proportion of samples were ID’d molecularly? Is it a proportion of those that were morphologically identified?

Yes. Species-specific proportions were used to compute HBR, CBR, EI and CBI estimates at the species level if ≥30 samples in the corresponding *Anopheles* Group were genotyped for each value of the grouping variable. The grouping variables were the collection time (06:00 pm to 06:00 am), the collection method (HCL or CBT) or the location of the mosquito collector (indoors or outdoors). For example, the number of *An. maculatus* ( *s.l.*) collected by HLC indoors and outdoors was 2240 and 5041 respectively. The proportion of *An. maculatus* ( *s.s.*) in the indoor and outdoor populations was 0.34 (133/392) and 0.37 (404/1084) respectively. The predicted number of *An. maculatus* ( *s.s.*) collected by HLC indoors and outdoors was 762 and 1865 respectively. The EI estimate for *An. maculatus *( *s.s.*) was therefore 0.71 (1865/2627). More detail were added in the methods section.

- Figure 5 – what are the dashed lines for? It might be more useful to use the line to show a community mean index

Vertical lines have been modified to address reviewer’s suggestion. The solid red line represents the mean index for *Anopheles spp.* mosquitoes, corresponding CIs were too narrow to be shown.

- Typo under “residual malaria transmission”: “35% of infection mosquitoes collected indoors between 9pm and 5pm”

The typo has been corrected.

- Rephrase the final sentence “in order to address specific problematic in the context of malaria elimination”

The final sentence has been changed to “the modalities of vector-control should be retuned to address problematic specific to malaria elimination”.

# Peer review: Reviewer response for version 1

**DOI:** 10.21956/wellcomeopenres.16087.r33968

**Published:** 2018-10-17

**Authors:** Catherine Bourgouin

**Affiliations:** 1 Functional Genetics of Infectious Diseases, Institut Pasteur, CNRS UMR 2000, Paris, France

The objective of the work presented by Chaumeau *et al* was aimed at further describing the entomological determinants of malaria transmission at the Myanmar border with Thailand, in order to guide the policy makers for malaria elimination in this region of low transmission.

 The investigation involved a longitudinal survey in four villages, using mosquito collection by Human Landing Catch as well as Cow Landing Catch over 21 months. The manuscript reports on a tremendous amount of work for both collecting mosquitoes, mosquito identification and Plasmodium detection in those mosquitoes by PCR. The data presented provided sound results in line with the objective.

 Nevertheless, several parts would benefit from clarification or simplification, while other information, for instance on Asian malaria vectors, would be valuable for readers who are not specialists in malaria transmission in Asia. See specific comments below.

 Additionally, I was expecting some deeper analysis or comments on possible differences among the 4 villages investigated and whether the ecology of the villages and the mosquito larvae could be included in malaria control recommendation.

 For the global analysis of the entomological indexes for malaria, I have found difficulties in following the calculation of HBR, SI and EIR, when related to the comparison of the 4 villages and the impact of the season (Table 4 and Table 5).

 The overall discussion could be better focused 1) on the global data, 2) the specificity or not of the transmission in the 4 villages to finish with stronger arguments for recommendation than the current discussion that sounds rather dogmatic on the specificity of transmission in Asia by mostly exophagic mosquitoes….

 Detailed comments:

**Title: “**Entomological determinants of malaria transmission in **Kayin** state, Eastern Myanmar: A **24-**month longitudinal study in **four** villages”

 The Methods mention a 21 months survey, that indeed might cover 2 years. As the survey in the 4 villages are not fully superimposed, it might be wise to describe the overlap for a better comprehensive value of the data.

 The “Kayin” state is barely mentioned in the report, bringing confusion when done ie: appearing only on page 13 beside the title and the abstract with “Therefore, baseline intensity of malaria transmission in hotspot villages from the Kayin state is likely to be higher than that reported in this study”

 Lastly, if there is no real discussion on the difference similarity among the 4 villages, why attracting with the “4 villages” in the title?

**Abstract: ** Some revision could be done if the authors agree on the following comments:

**Methods**

**Study sites: ** Providing the global description of the villages, as done in the J. Vector ecology Paper (ref14), would save time, rather than going to the published document as well as possibly useful for the discussion. Was the MDA still active during the whole entomological survey?

**Entomological surveys: **Were the house randomly chosen? Were the preliminary data (ref14) included in the analysis? Consider simplification:  instead “6 sites” use 5 houses plus one cow collection. Verify the accuracy of the sentence for mosquito collection under the net covering the cow.

**Data analysis:** Expressing SI / 1000 mosquito can be confusing. I would keep with the classical SI expressed as %. The following sentence “Entomological inoculation rate (EIR) was defined as the number of positive in qrtPCR Plasmodium divided by the corresponding number of person-nights of collection, adjusted over the proportion of collected mosquitoes that were analyzed by qrtPCR Plasmodium” is fully unclear to me. What do you mean by “adjusted over the proportion of collected mosquitoes that were analyzed by qrtPCR Plasmodium”? Again, why not keeping with the classical definition of EIR: HBR * SI%/100.

 The CI calculation might need approval from an external statistician.

**Results:**

**Biodiversity of the Anopheles entomo-fauna**

 From my understanding of the Methods, the total person-nights should be 4200 and total cow-nights 420. Did I miss something?

 “Potential malaria vectors”: could you provide your definition of those? A species table as sup data could be useful for non-specialist of Asian mosquito vectors. In figure 2 “other species” what does this include?

 Fig2: why not commenting the comparative results for HLC and CBC and among the villages. Homogenize between fig 2 A, Legend, Table 1 and main text : CBC or CBT.

 “Results of the molecular identification are presented in the Table 1.”

**Malaria vectors**

 Table 2: Correct Mean Pf-EIR for Funestus : 0.1, not 0.01. Also in this table and followings, could not really follow the calculation from HBR and SI to the EIR, from the values of the former indexes, possibly as the mean values were calculated across the different months and/or villages. This might need to be specify at some point.

 It would be also informative to provide the raw data as supp file for the identification of the species for the Plasmodium positive samples.

 Sporozoite quantification: It is indeed interesting to be able to quantitate the sporozoite load for each positive mosquito. I would suggest to specify in the sup file that the calculated LOD of  6 Pf sporo or 4 Pv sporo are indeed per mosquito, according to your method.

 For this load quantification data, I am not sure that any correlation with oocyst detection can be made. There is, to my knowledge, no method to determine in field collected mosquitoes that the oocysts one detects are the one providing the detected sporozoites in head-thoraxes. They might come from a secondary infection. I nevertheless agree that in very low transmission area the probability for a mosquito to feed twice on a gametocyte carrier is rather very low. In addition, please indicate the oocyst detection method, PCR? And how the midgut were preserved?

 Table 3 could be placed as supp data.

**Host-seeking behaviour of Anopheles mosquitoes**

 First sentence: “overall”, be more precise as taking into account all mosquitoes captured by HLC…

 Nevertheless, again I cannot obtain the same numbers for both HBR and CBR, though very closed.

 Paragraph on Zoophagy : I am not sure that a zoophagic index can indeed be calculated as the surface of a cow exceed the surface of the human skin for HLC provided by 10 persons , plus volume of air and odors and containment of the cow. This is my opinion on this, but preferentially zoophagic/anthropophagic is OK.

 Outdoor and early biting paragraph: Figure 6 and 7: revised the labeling for Fig 6, no distinction in the grey colours. Although there is a tendency for early biting it does not stand for all species ie minimus.

 Globally there is far too much figures for this section. Relocating some to the sup data will save space for the next section, that is at the center of the question: who is transmitting and when?

**Entomological indices of malaria transmission**

 As said above, this might be the most important analysis. However, I still have some difficulties with the calculation in Table 4 and 5, At what level were the mean calculated?

 Because you have all these nice data as monthly collection (see sup file 1) , why not comparing HBR per species (or even group) and EIR for each village, over time. This will clearly show who and when most transmission occurs  and easier to visualize as graphs rather than tables as Table 4 and 5.

**Discussion:**

 In my general comment I already mentioned that the discussion could benefit from a better focus.

 I would add on the argument Pf and Pv transmission and season (second paragraph) that one needs to keep in mind, that beside having mosquitoes, gametocytes are also needed and Pv carriers who are not totally cured are excellent providers of gametocytes the year around. It is why it is also important that in the Methods section is included whether MDA was on or not and how Pv carriers were treated.

 Also this sentence might be strongest :” In this study, only 35% of the mosquitoes infected with Plasmodium were collected indoors between 09:00 pm and 05:00 pm, because of outdoor and early biters”, (page 14)., THE TIMING When people do not sleep under a net. The 35% appears as 36% in figure 8 : correct?

 Lastly: the last sentence of this paragraph is unclear : “Consequently, the paradigm of residual transmission as experienced in high transmission settings of Africa does not apply to the Thailand-Myanmar border area and a drastic shift in vector-control interventions is required.”

 Could you explain what is the paradigm?

I have read this submission. I believe that I have an appropriate level of expertise to confirm that it is of an acceptable scientific standard, however I have significant reservations, as outlined above.

# Response

**Published:** 2019-01-27

**Authors:** Victor Chaumeau

**Affiliations:** Shoklo Malaria Research Unit, Thailand

We are thankful to the reviewer for her useful feed-back on the manuscript. The manuscript has been edited as per reviewer’s suggestions in order to improve the clarity of this report and to provide the reader with more background information on Southeast Asian malaria vectors. We agree with the reviewer that a more detailed explanatory analysis on the heterogeneity of malaria transmission in the four villages would have been valuable. Additional figures were added in the Supplementary File 1 in order to better show the differences in malaria transmission in the four villages. However, in the absence of appropriate datasets, the mechanisms by which local ecological factors and larval habitat interact with malaria transmission cannot be explained. Noteworthy, the primary objective of this paper was not to point out specific differences in particular study villages, but instead to identify key aspects of malaria transmission that are relevant to the Kayin state and other areas in Southeast Asia. Raw numbers used for the calculation of the entomological indices estimates were added in the corresponding tables, additional references on the method used for the calculation of entomological inoculation rate were added in the Methods section. Regarding reviewer’s suggestion on the discussion, we wish to keep the outline of the manuscript version 1. Although a comparison of the entomological indices in different geographic areas and across the endemicity spectrum would be of great interest, a review of the global data seems beyond the scope of the current paper as it would represent a tremendous amount of work and could be the object of a full review article. In addition, the specificities of malaria transmission in the 4 villages (and Southeast Asian malaria transmission settings) were discussed into details in the first version of the manuscript. The point that were discussed included the low infection rates in malaria vectors and high biting rates, the seasonality in *Plasmodium falciparum* transmission, the heterogeneity of the entomological indices, the low parasite loads in infected vectors, the high diversity of malaria mosquitoes, their outdoor and early biting pattern, their opportunistic host type selection and ability to feed on livestock’s. Finally, clear recommendations for malaria vector-control in this region were made in a specific paragraph using our results as a rational: we suggested to evaluate the use of outdoor spraying of insecticides on daytime resting habitats to tackle exophilic mosquitoes and veterinary approaches to tackle zoophagic malaria vectors. Regarding reviewer’s inquiry about larval source management, it is difficult for us to discuss this intervention because we did not collect data on larval stages. Existing bibliography and our data on adults suggest that larval habitats are diverse, fragmented and poorly accessible, which probably limits the feasibility of larval source management in Kayin state. Regarding the use of “village ecology” as a vector-control tool, it is unclear to us what set of interventions the reviewer was referring to. What may appear to be “dogmatic” for the reviewer ( *i.e.* the specificity of transmission in Asia by mostly exophagic mosquitoes) may be considered by others as one of the main factor explaining the differences in mosquito bed-nets and indoor residual spraying outcomes in Southeast Asia when compared to Africa. In the current context of malaria elimination, we therefore believe that this notion should be at the center of the discussion. Point-to-point answers to specific comments are given below:

- The Methods mention a 21 months survey, that indeed might cover 2 years. As the survey in the 4 villages are not fully superimposed, it might be wise to describe the overlap for a better comprehensive value of the data.

Reviewer’s suggestion has been addressed by presenting the collection schedule in the Table S1 of the revised manuscript.

- The “Kayin” state is barely mentioned in the report, bringing confusion when done ie: appearing only on page 13 beside the title and the abstract with “Therefore, baseline intensity of malaria transmission in hotspot villages from the Kayin state is likely to be higher than that reported in this study”

Reviewer’s concern was addressed by changing “Thailand-Myanmar border” to Kayin state were possible and by mentioning more often “Kayin state” in the main text.

- Lastly, if there is no real discussion on the difference similarity among the 4 villages, why attracting with the “4 villages” in the title?

We agree with the reviewer that a more detailed explanatory analysis on the heterogeneity of malaria transmission in the four villages would have been valuable. However, in the absence of appropriate datasets, this analysis is not possible. Documenting differences is particular villages was not the only objective of including 4 villages in the study design. Extensive longitudinal follow-up in four villages has also allowed us to identify key aspects of malaria entomology that would not have been accurately describe with less sites or time-point collections. Therefore, we think it is important to mention the duration of the follow-up and the number of villages in the title.

- Abstract:  Some revision could be done if the authors agree on the following comments:

The abstract was modified as per reviewer’s suggestions.

- Providing the global description of the villages, as done in the J. Vector ecology Paper (ref14), would save time, rather than going to the published document as well as possibly useful for the discussion. 

Reviewer’s comment was addressed by adding more details in the paragraph “Study villages” of the Methods section.

- Was the MDA still active during the whole entomological survey?

The reviewer is right to inquire about the timing of mass antimalarial drug administration and to stress that the elimination intervention has been poorly described in the manuscript. As specific paragraph describing the intervention has been added to the Methods section and additional references were provided to the reader.

- Were the house randomly chosen?

Yes, the houses were randomly selected and this has been stated in the revised manuscript.

- Were the preliminary data (ref14) included in the analysis?

Yes, the preliminary data were included in the analysis.

- Consider simplification:  instead “6 sites” use 5 houses plus one cow collection.

The phrasing of this sentence has been simplified as per reviewer’s suggestion.

- Verify the accuracy of the sentence for mosquito collection under the net covering the cow.

The accuracy of the sentence has been verified.

- Expressing SI / 1000 mosquito can be confusing. I would keep with the classical SI expressed as %.

We agree with the reviewer. SI values were expressed as percentages in the revised manuscript.

- The following sentence “Entomological inoculation rate (EIR) was defined as the number of positive in qrtPCR Plasmodium divided by the corresponding number of person-nights of collection, adjusted over the proportion of collected mosquitoes that were analyzed by qrtPCR Plasmodium” is fully unclear to me. What do you mean by “adjusted over the proportion of collected mosquitoes that were analyzed by qrtPCR Plasmodium”? Again, why not keeping with the classical definition of EIR: HBR * SI%/100.

This definition of EIR is based on the work of Drakeley *et al.* 2003 (An estimation of the entomological inoculation rate for Ifakara: a semi-urban area in a region of intense malaria transmission in Tanzania), also reviewed by Tusting *et al.* 2014 (Measuring Changes in Plasmodium falciparum Transmission: Precision, Accuracy and Costs of Metrics). When EIR is analysed as the product of SI per HBR (equation 1), computing confidence intervals (CIs) becomes difficult since HBR and SI are not independent of each other; CIs can be computed more easily when analyzing EIR as a biting rate (taking into account only the sporozoite-infected mosquitoes) (equation 2). Adjusting the number of person-nights of collection over the proportion of collected mosquitoes that were analyzed by qrtPCR allow to take into account that not all mosquitoes were screened for Plasmodium infection (equation 3). Of note, this adjustment makes the calculation equivalent to the “classical” definition of EIR (equation 4) and has little impact on the estimate value as 99% (50082/50708) of the collected specimens were analysed by PCR Plasmodium.

 (1)  EIR=SI ×EIR

 (2)  EIR=(Nb.  sporozoite positive mosquitoes )/(Nb.person.nights of collection)

 (3)  EIR=(Nb.  sporozoite positive mosquitoes )/(Nb.person.nights of collection×(Nb.of mosquitoes tested by PCR "/" Nb.of collected specimens))

 (4)  EIR=(Nb.  sporozoite positive mosquitoes )/(Nb.of mosquitoes tested by PCR )  ×  (Nb.of collected specimens)/(Nb.person.nights of collection)

- The CI calculation might need approval from an external statistician.

The method used for the CI calculation in this paper was also reviewed in Landier *et al.* WOR, 2017 and Chaumeau *et al.* JID, 2018.

- From my understanding of the Methods, the total person-nights should be 4200 and total cow-nights 420. Did I miss something ?

We are thankful to the reviewer for pointing this ambiguity. Exceptions to the initial study design have been explicitly mentioned in the corresponding paragraph of the Methods section. The number of person-nights and cow-nights for each survey is also presented in the Table S1 of the revised manuscript.

- “Potential malaria vectors”: could you provide your definition of those?

We are thankful to the reviewer for pointing this unclear wording, it has been removed from the revised manuscript.

- A species table as sup data could be useful for non-specialist of Asian mosquito vectors.

Reviewer’s suggestion was addressed by adding a species table in the Introduction.

- In figure 2 “other species” what does this include?

The legend of Figure 2 has been reworded. “Potential malaria vectors” has been changed to “ *a priori* malaria vectors”, which refers to groups of *Anopheles* species that have been previously found infected with human malaria parasite in other studies (Funestus, Maculatus, Leucosphyrus, Barbirostris and Annularis Groups). “Other species” has been changed to “other groups” and includes all the other groups that have been reported in the paragraph “ *Anopheles* diversity”. More details were also added in the legend note.

- Fig2: why not commenting the comparative results for HLC and CBC and among the villages.

Ranges were cited in the main text to point village differences in the species distribution at the group level. Moreover, differences in species-specific distribution between villages assessed by molecular identification were commented into details in the main text and are reported in the Supplementary File 1, Table S2.

- Homogenize between fig 2 A, Legend, Table 1 and main text : CBC or CBT.

Figure and table captions, and in-text citations were harmonized to CBT in the revised manuscript.

- Table 2: Correct Mean Pf-EIR for Funestus : 0.1, not 0.01.

Mean Pf-EIR for Funestus Group has been corrected.

- Also in this table and followings, could not really follow the calculation from HBR and SI to the EIR, from the values of the former indexes, possibly as the mean values were calculated across the different months and/or villages. This might need to be specify at some point.

The raw number used for the calculation was provided in all tables. Unless specified otherwise, the calculation was made using the entire dataset.

- It would be also informative to provide the raw data as supp file for the identification of the species for the Plasmodium positive samples.

Reviewer’s suggestion was addressed by presenting the raw data of identification of the species for *Plasmodium* positive samples in the Supplementary File 1, Table S4.

- I would suggest to specify in the sup file that the calculated LOD of  6 Pf sporo or 4 Pv sporo are indeed per mosquito, according to your method.

Reviewer’s suggestion has been added to the revised Supplementary File 2.

-  For this load quantification data, I am not sure that any correlation with oocyst detection can be made. There is, to my knowledge, no method to determine in field collected mosquitoes that the oocysts one detects are the one providing the detected sporozoites in head-thoraxes. They might come from a secondary infection. I nevertheless agree that in very low transmission area the probability for a mosquito to feed twice on a gametocyte carrier is rather very low.

We are thankful to the reviewer for pointing that the correlation between oocysts and sporozoite densities is not straightforward, especially in wild mosquitoes, and that it may yield to spurious interpretation. The correlation between oocysts and sporozoite densities has been removed from the revised manuscript.

- In addition, please indicate the oocyst detection method, PCR? And how the midgut were preserved?

A statement on oocysts detection method (PCR) and midgut preservation has been added in the Methods section of the revised manuscript in order to address reviewer’s comment.

- Table 3 could be placed as supp data.

Table 3 has been moved to supplemental data as per reviewer suggestion.

- First sentence: “overall”, be more precise as taking into account all mosquitoes captured by HLC…

We are thankful to the reviewer for pointing this inaccurate wording. The word “overall” has been changed to “taking into account the whole dataset”.

- Nevertheless, again I cannot obtain the same numbers for both HBR and CBR, though very closed.

We hope that the above explanations will help the reader in understanding the calculation used for the entomological indices.

- I am not sure that a zoophagic index can indeed be calculated as the surface of a cow exceed the surface of the human skin for HLC provided by 10 persons , plus volume of air and odors and containment of the cow. This is my opinion on this, but preferentially zoophagic/anthropophagic is OK.

We are thankful to the reviewer for mentioning that estimating zoophagy accurately would require a different approach than that used in our study, although comparing the human- and cow-biting rates as an index can be useful to describe the capacity of some specie to feed on bovines. The wording “zoophagic index” has been changed to “cow-biting index” (CBI) to address reviewer’s comment.

- Figure 6 and 7: revised the labeling for Fig 6, no distinction in the grey colours.

Distinction in the grey colours is shown in the Key box in the figure (figure S12 of the revised manuscript).

- Although there is a tendency for early biting it does not stand for all species ie minimus.

“ *Anopheles* mosquitoes exhibited an outdoor and early biting pattern” has been changed to “ *Anopheles* mosquitoes exhibited an outdoor and/or early biting pattern” in order to address reviewer’s comment. Although the biting rate of *An. minimus* did not peak during the early evening/morning, *An. minimus* biting rate between 5-6 pm and 5-6 am was higher than that of any other *Anopheles* species. Moreover, the high biting rates observed at 5-6 pm and 5-6 am suggest that this species was active before and after the standard collection time, which would meet the definition of early biting. 

- Globally there is far too much figures for this section. Relocating some to the sup data will save space for the next section, that is at the center of the question: who is transmitting and when?

Some figure from this section were relocated in the supplementary file as per reviewer’s suggestions.

- As said above, this might be the most important analysis. However, I still have some difficulties with the calculation in Table 4 and 5, At what level were the mean calculated?

The mean was calculated using the entire dataset for the corresponding grouping variable (village in the table 4 and season in the table 5) using the definition provided in the Method section. Raw data used for the calculation of entomological indices estimates were added to all tables to help the reader understanding the details of the calculation.

- Because you have all these nice data as monthly collection (see sup file 1) , why not comparing HBR per species (or even group) and EIR for each village, over time. This will clearly show who and when most transmission occurs and easier to visualize as graphs rather than tables as Table 4 and 5.

The figures suggested by the reviewer were added in the Supplementary File 1, Figures S7-S10. We think that presenting mean values of entomological indices in tables 4 and 5 is important to understand malaria transmission in this area and compare with other settings, which would maybe not appear as clearly in the figures presenting the longitudinal data.

- In my general comment I already mentioned that the discussion could benefit from a better focus. I would add on the argument Pf and Pv transmission and season (second paragraph) that one needs to keep in mind, that beside having mosquitoes, gametocytes are also needed and Pv carriers who are not totally cured are excellent providers of gametocytes the year around. It is why it is also important that in the Methods section is included whether MDA was on or not and how Pv carriers were treated.

More details on the elimination intervention were added in the Intervention paragraph, Methods section and more reference were added in the revised version of the manuscript. We think that discussing the the dynamic of gametocyte carriage may be beyond the scope of this article. For example, Pf carriers are also likely to be excellent providers of gametocytes the year around if not treated. A more detailed analysis of the relationship between parasitological and entomological indices was published in the Journal of Infectious Diseases by Chaumeau *et al.* in 2018.

- Also this sentence might be strongest :” In this study, only 35% of the mosquitoes infected with *Plasmodium* were collected indoors between 09:00 pm and 05:00 pm, because of outdoor and early biters”, (page 14)., THE TIMING When people do not sleep under a net.

The typo 05:00pm has been changed to 05:00am. The window 09:00pm to 05:00am indoors is expected to cover the timing when people do sleep under a net. The stronger wording suggested by the reviewer was added to the revised version of the manuscript.

- The 35% appears as 36% in figure 8 : correct?

The in-text citation has been corrected.

- Lastly: the last sentence of this paragraph is unclear : “Consequently, the paradigm of residual transmission as experienced in high transmission settings of Africa does not apply to the Thailand-Myanmar border area and a drastic shift in vector-control interventions is required.” Could you explain what is the paradigm?

The paradigm is that – based on observations made in Sub-Saharan Africa - universal coverage with LLINs would prevent most of the infective bites, and conversely that the part of the transmission that would not be covered by LLINs would be only a residue ( *i.e.* a small part) of transmission at baseline. Some control or elimination programs indeed still rely mainly, and sometimes solely, on mass distribution of LLINs. The data presented in this study (as well as other references cited in the discussion) clearly demonstrate that LLINs have only a marginal impact on disease prevention in the GMS rather than preventing the main part of the transmission. Therefore, we do not think that is would be accurate to refer to the part of the transmission that is not prevented by LLINs as “residual transmission” in the transmission settings described in this study.
